# RNA‐Binding Protein RBM25 Targets the mRNA Stability of GTPase Rab22a to Restrict Viral Entry and Infection

**DOI:** 10.1002/advs.76160

**Published:** 2026-06-16

**Authors:** Yingying Ding, Huiying Chen, Yuyu Jiang, Chunyan Zhao, Jie Bai, Yan Xiang, Zeting Wang, Xixi Wang, Bing Rui, Wanda Tang, Yue Ding, Zhenzhen Zhan, Yunkai Zhang, Xingguang Liu

**Affiliations:** ^1^ National Key Laboratory of Immunity & Inflammation Department of Pathogen Biology Naval Medical University Shanghai China; ^2^ Key Laboratory of Biological Defense Ministry of Education Shanghai China; ^3^ Naval Medical Center Naval Medical University Shanghai China; ^4^ Department of Microbiology Naval Medical University Shanghai China; ^5^ Shanghai Institute of Transplantation Renji Hospital Shanghai Jiao Tong University School of Medicine Shanghai China

**Keywords:** antiviral immunity, mRNA stability, Rab22a, RBM25, RC3H1, respiratory virus, viral entry

## Abstract

Viral infections trigger complex host defense responses, yet many key regulatory mechanisms remain undefined. Here, we identify the RNA‐binding protein RBM25 as a potent, broad‐spectrum host antiviral factor, independently of the type I interferon (IFN‐I) pathway. Viral infection downregulates RBM25 expression, and RBM25‐deficient mice exhibit enhanced susceptibility to multiple viruses and more aggravated tissue damage. In vitro, RBM25 inhibits the viral infection and replication across a spectrum of RNA and DNA viruses. Mechanistically, the antiviral activity of RBM25 is independent of IFN‐I signaling and is instead linked to an early blockade in the viral life cycle. RBM25 specifically impedes viral cell entry through the suppression of the host GTPase Rab22a, a well‐known facilitator of viral endocytosis. Virus infection‐elicited downregulation of RBM25 results in Rab22a upregulation, which consequently potentiates viral entry. Furthermore, we elucidate the post‐transcriptional mechanisms that RBM25 interacts with RC3H1 (ring finger and CCCH‐type domains 1) to form an RNA‐binding complex that binds and destabilizes *Rab22a* mRNA, thereby limiting its protein translation. Collectively, our work unveils the RBM25/RC3H1‐Rab22a axis as an interferon‐independent post‐transcriptional pathway that governs viral entry by modulating the mRNA stability of a critical host endocytosis factor, which presents a potential target for developing broad‐spectrum antiviral strategies.

## Graphical abstract

This study identifies RNA‐binding protein RBM25 as a broad‐spectrum antiviral factor acting independently of type I interferon. It blocks viral entry by suppressing GTPase Rab22a via the RC3H1‐mediated destabilization of *Rab22a* mRNA. Viral downregulation of RBM25 enhances GTPase Rab22a expression and viral entry, revealing an unreported post‐transcriptional pathway for viral immune evasion.

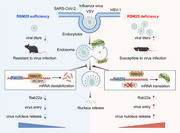

## 1 Introduction

Viral infections pose a persistent threat to global public health, driven by emerging and re‐emerging pathogens‐such as influenza A Virus (IAV) [1, 2, 3] and severe acute respiratory syndrome coronavirus 2 (SARS‐CoV‐2) that hijack host cellular machinery [4]. Viral replication exploits a sequential series of host processes, including receptor attachment, cell entry (via endocytosis, membrane fusion, or pore formation), intracellular trafficking, genome replication/transcription, translation, assembly, and egress [5]. Among these, viral entry is a critical rate‐limiting step that determines host tropism and pathogenicity; multiple RNA viruses, including IAV [6], SARS‐CoV‐2 [7], and Vesicular Astomatitis Virus (VSV) [8], rely on conserved endocytic pathways and trafficking machinery to access the cytoplasm or nucleus [9]. Infection typically begins with receptor binding, followed by receptor‐mediated endocytosis‐often through clathrin‐coated pits. The internalized virus traffics through the endosomal system, where the acidic environment and proteases in early or late endosomes trigger conformational changes in viral structures, leading to membrane fusion or pore formation and release of the viral genome [10]. For instance, IAV and SARS‐CoV‐2 fuse primarily in late endosomes, whereas Middle East respiratory syndrome coronavirus (MERS‐CoV) fuses preferentially in early endosomes [11, 12].

The endocytic route represents a key vulnerability point for host defense, as the precise timing and location of fusion critically influence viral infectivity and host range. Host cells have evolved intrinsic restriction factors that target specific steps of the viral life cycle, with type I interferon (IFN‐I)‐independent factors acting early in infection (prior to IFN‐I induction) emerging as crucial modulators of entry‐particularly for viruses that evade IFN‐I responses [13, 14]. Nevertheless, the host factors that restrict viral entry through IFN‐I‐independent mechanisms, and their molecular interplay with viral entry machinery, remain incompletely defined, representing a critical gap in viral biology and a barrier to the development of broad‐spectrum antiviral strategies.

RNA‐binding proteins (RBPs) are a family of more than 4200 human members that govern RNA metabolism (such as alternative splicing, nucleus transport, translation, and decay) via specific recognition of RNA sequences [15], for instance, AU‐rich elements and CpG motifs or structural features including stem‐loops. RBPs have emerged as the central mediators of host‐virus crosstalk, either restricting viral life cycle steps or being co‐opted by viruses to promote replication. Key examples include: (i) Direct viral RNA restriction: zinc finger antiviral Protein (ZAP) binds CpG‐rich RNAs (e.g., human immunodeficiency virus type 1 (HIV‐1), Sindbis virus) to recruit the RNA exosome [16]; RNA‐activated protein kinase (PKR) recognizes replicative double‐stranded RNAs (dsRNAs) (e.g., flaviviruses, coronaviruses) to inhibit translation [17]; TIA‐1/TIAR sequester viral RNAs in stress granules [18]. (ii) Viral hijacking: human antigen R (HuR) stabilizes HCV/enterovirus RNAs via 3’ untranslated region (3’UTR) binding [19]; heterogenous nuclear ribonucleoprotein K (hnRNPK) aids HIV‐1 RNA packaging [20]. (iii) Regulation of viral dependency factors: DEAD‐Box Helicase 3 X‐Linked (DDX3X) modulates host translation and viral RNA trafficking [21]. Despite the extensive repertoire of RNA‐binding proteins (RBPs) identified in viral pathogenesis, a significant gap persists: relatively few RBPs have been implicated in the earliest step of the viral lifecycle‐viral entry. The majority of characterized RBPs tend to exert their regulatory effects at post‐entry stages, such as viral replication and translation. In contrast, entry restriction is predominantly mediated by membrane‐associated proteins (e.g., IFITM3, which modulates endosomal fluidity) or trafficking regulators (e.g., Rab GTPases, which govern endosome maturation) [22, 23]. Notably, no RBPs have yet been clearly identified to directly modulate viral entry through post‐transcriptional regulation of the host entry machinery. This highlights a critical unmet need in the field, particularly given the evolutionary conservation of viral entry mechanisms across diverse pathogens.

RNA Binding Motif 25 (RBM25), also termed Snu71 in budding yeast, was initially identified as a U1 small nuclear ribonucleoprotein that functions in early spliceosome formation. Aberrant expression or activity of RBM25 increases the host susceptibility to tumorigenesis, cardiovascular diseases, and Alzheimer's disease [24, 25, 26]. Our lab previously revealed that RBM25 acts as the pre‐mRNA splicing regulator of glycolytic enzyme ATP‐Citrate Lyase for rewiring inflammatory macrophage metabolism during autoimmunity and aging‐associated immunological disorders [27]. However, the potential role of RBM25 in viral entry and infection, particularly its interaction with viral entry machinery or regulation of the viral life cycle, has not been explored. In this study, we identify RBM25 as a novel broad‐spectrum host antiviral factor. RBM25 restricts viral infection by blocking viral entry independently of the canonical TANK binding kinase 1 (TBK1) interferon regulatory factor 3 (IRF3) pathway. Instead, RBM25 specifically interacts with RC3H1, an RBP involved in mRNA stability control, to form a complex that binds to *Rab22a* mRNA and promotes its degradation, thereby limiting Rab22a‐dependent viral entry. Together, our findings unveil the common immune evasion pathway of innate antiviral response by hijacking host RBM25 expression and propose an RBM25/RC3H1–Rab22a axis that functions as an IFN‐independent intrinsic pathway controlling viral entry. This work expands the molecular understanding of RBP‐mediated host antiviral defense and highlights a potential target for developing broad‐spectrum antiviral strategies.

## 2 Results

### 2.1 Decreased Expression of RBM25 Following Multiple Virus Infection

**FIGURE 1:**
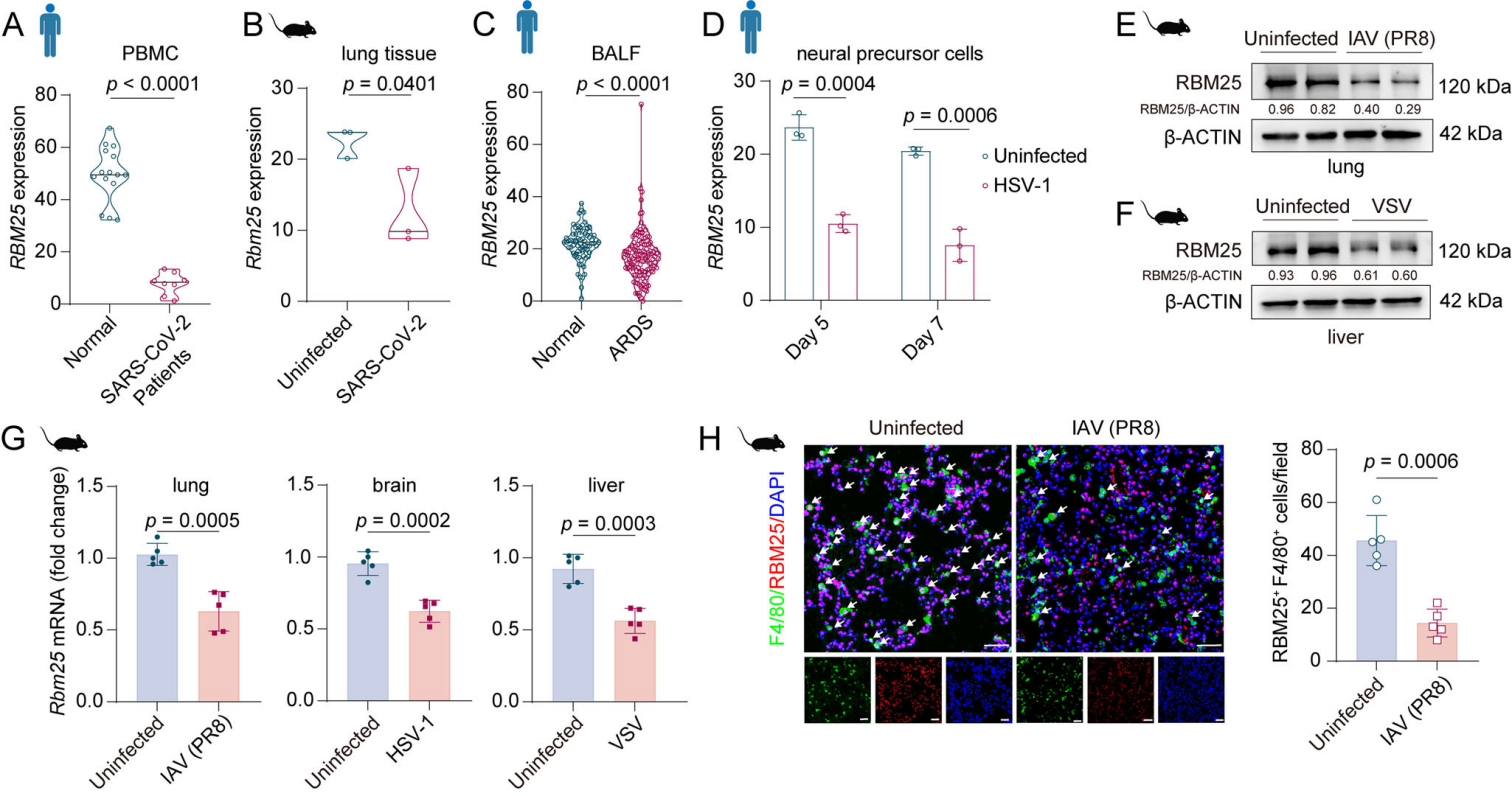
Decreased expression of RBM25 following viral infection. (A) Violin‐plots of RBM25 expression in human PBMCs from public microarray datasets (GEO: GSE215262; SARS‐CoV‐2 infected patients, *n* = 8; normal controls, *n* = 15). (B) Violin‐plots of *Rbm25* expression in mice lung tissues from public microarray datasets (GEO: GSE223056; SARS‐CoV‐2 infected mice, *n* = 3; uninfected mice, *n* = 3). (C) Violin‐plots of RBM25 expression in human BALF cells from public microarray datasets (GEO: GSE279069; ARDS patients caused by the respiratory viruses, *n* = 135; normal, *n* = 74). (D) Scatterplots of RBM25 expression in human neural precursor cells infected with HSV‐1 for 5 days or 7 days from public microarray datasets (GEO: GSE236646; infected human neural precursor cells, *n* = 3; uninfected control, *n* = 3). (E) Immunoblots analysis of RBM25 in lung tissues of mice infected with PR8 influenza virus or treated with PBS. β‐ACTIN was used as a loading control. (F) Immunoblots analysis of RBM25 in liver tissues of mice infected with VSV or treated with PBS. β‐ACTIN was used as a loading control. (G) RT‐qPCR analysis of *Rbm25* mRNA levels in lung tissues from mice infected with PR8 influenza virus or in brain tissues from mice infected with HSV‐1 or in liver tissues from mice infected with VSV, respectively (*n* = 5 per group). (H) Representative immunofluorescence (IF) images of F4/80 (green) and RBM25 (red) expression in lung sections from WT mice on day 7 after PR8 influenza virus infection or PBS treatment (scale bar, 50 µm). Data are presented as mean or mean ± SD. Two‐tailed unpaired Student's *t* test (A–D, G, H).

To identify the potential host factors associated with innate antiviral responses, we first analyzed the expression levels of the genes encoding RBPs in peripheral blood mononuclear cells (PBMCs) from SARS‐CoV‐2‐infected patients and healthy controls using publicly available microarray datasets. Compared to healthy controls, the expression of some RBP family members remained unchanged in PBMCs from SARS‐CoV‐2‐infected patients, while others were dramatically reduced following SARS‐CoV‐2 infection. Among these, RBM25 exhibited the most pronounced decrease (Figure 1A and Figure S1A–C). Similarly, *RBM25* expression was significantly inhibited in lung tissues of SARS‐CoV‐2‐infected mice, compared to controls (Figure 1B). Moreover, reduced RBM25 expression was also observed in bronchoalveolar lavage fluid (BALF) cells from patients with acute respiratory distress syndrome (ARDS) caused by respiratory viral infections (Figure 1C), as well as in neural precursor cells (NPCs) infected with Herpes simplex virus 1 (HSV‐1) (Figure 1D).

Next, we established the IAV and vesicular stomatitis virus (VSV) infection animal model to investigate the dynamic expression of RBM25. Protein levels of RBM25 were also markedly reduced in the lung of mice infected with the influenza virus (PR8 Strain) or liver tissues of mice infected with VSV, respectively (Figure 1E,F). Consistently, mRNA expression of RBM25 was significantly decreased in the IAV‐infected lung, HSV‐1 infected brain, or VSV‐infected liver tissues of mice, respectively (Figure 1G). Furthermore, both protein and mRNA levels of RBM25 were inhibited in mouse peritoneal macrophages infected with IAV (PR8), HSV‐1, or VSV in dose‐ and time‐dependent manner, determined by different multiplicity of infection (MOI) and different infection periods (Figure S1D–F). In addition, no significant change of RBM25 expression was observed in macrophages with poly (I:C) transfection or ultraviolet‐inactivated VSV (UV‐VSV, which lacks replicative activity) treatment (Figure S1G,H), indicating that the downregulation of RBM25 is dependent on alive invading virus. To further assess the dynamic pattern of intracellular RBM25 expression during IAV infection, we evaluated its expression in lung tissues from IAV‐infected and control mice. Immunofluorescent (IF) staining of macrophages from lung tissue of IAV (PR8) infected mice showed that RBM25 expression was significantly reduced in F4/80^+^ macrophages (Figure 1H). Collectively, the consistent decreased expression of RBM25 was observed across a range of RNA and DNA viruses infection, both in vivo and in vitro. These data indicate that host RBM25 might display the potential role of antiviral infection.

### 2.2 Genetic Ablation of RBM25 in Macrophages Increases the Susceptibility to IAV, HSV‐1 and VSV Infection

**FIGURE 2:**
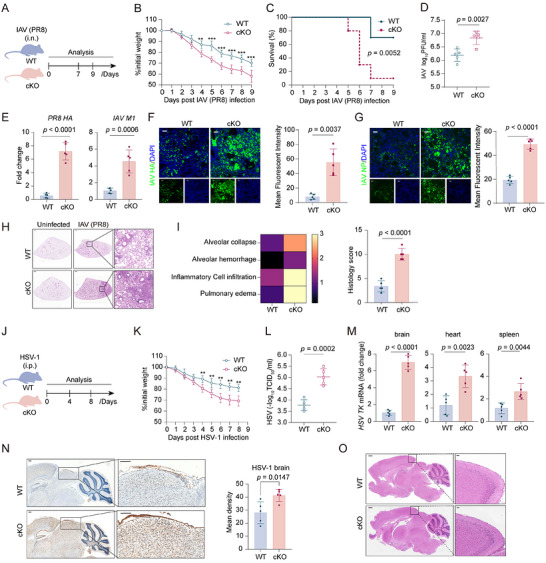
RBM25 deficiency results in greater susceptibility to IAV and HSV‐1 infection. (A) *Rbm25*‐cKO and WT mice were intranasally (*i.n*.) inoculated with PR8 influenza virus. (B) Body weight of *Rbm25*‐cKO and WT mice infected with a lethal dose of PR8 influenza virus (120 PFU) were monitored daily (*n* = 6 per group). ^**^ *p* < 0.01, ^***^ *p* < 0.001. (C) Survival of *Rbm25*‐cKO and WT mice treated as in (A) (*n* = 10 per group). Kaplan‐Meier survival curves were compared using log‐rank (Mantel‐Cox) analysis. (D) Viral titers in lung tissues from mice infected with a sublethal dose (50 PFU., *n* = 5 per group) at 7 dpi. (E) RT‐qPCR analysis of *PR8 HA* and *IAV M1* mRNA in lung tissues from mice as in (D). (F, G) IF analysis of IAV HA (F) and IAV NP (G) expression in lung tissues from mice, as in (D). The mean fluorescent intensity (MFI) of corresponding IF images were calculated by Image J software. Scale bar, 30µm. (H) Representative micrographs of lung histology stained with H&E from mice as in (D). Scale bar, 500 µm (left), 50 µm (right). (I) Lung histology scoring of *Rbm25*‐cKO and WT mice treated as in (H), *n* = 5 per group. (J) *Rbm25*‐cKO and WT mice were *i.p*. infected with HSV‐1. (K) Body weight of *Rbm25*‐cKO and WT mice infected with HSV‐1 (5 × 10^8^ PFU) was monitored over time (*n* = 5 per group). ^**^ *p* < 0.01. (L) HSV‐1 titers in the brain from *Rbm25*‐cKO and WT mice at day 4 after treatment, as in (K). (M) RT‐qPCR analysis of *HSV‐1 TK* mRNA in the brain, heart, or spleen tissues from *Rbm25*‐cKO and WT mice at day 4 after treatment as in (K). (N) Representative image of IHC for the whole brain in a sagittal section of *Rbm25*‐cKO and WT mice, as in (K), by staining the antibody against HSV‐1 gB (scale bars, 200 µm), *n* = 5 mice per group. The mean density was calculated by Image J software. (O) H&E staining of the whole brain in a sagittal section of *Rbm25*‐cKO and WT mice, as in (K). Scale bar, 500 µm (left), 50 µm (right). Data are presented as the mean ± SD. Unpaired two‐tailed Student's *t*‐test (B, D–G, I, K–N).

Considering RBM25 expression was dramatically suppressed during virus infection, especially in F4/80^+^ macrophages, we next examined whether genetic depletion of RBM25 in macrophages affected host innate antiviral responses in mice. Therefore, macrophage‐specific *Rbm25* knockout mice (*Rbm25* ^flox/flox^ *Lyz2* ^cre+^, hereinafter referred to *Rbm25*‐cKO mice) were generated, with the deficiency of RBM25 in peritoneal and bone marrow‐derived macrophages (BMDMs), as compared to WT littermates (*Rbm25* ^flox/flox^, hereinafter defined as WT mice) (Figure S2A,B). Consistent with previous findings [27], under steady‐state conditions, *Rbm25*‐cKO mice exhibited normal development and differentiation of key antiviral immune cells in PBMCs, livers, and spleens, including myeloid subsets (neutrophils, monocytes, macrophages, pDCs, Figure S2C–E.) and lymphoid lineages (T cells, B cells, NK cells, Figure S3). To further evaluate the important role of RBM25 in host defense to viral infection, *Rbm25*‐cKO mice and WT littermates were challenged with a panel of pathogenic viruses. First, *Rbm25*‐cKO and WT mice were intranasally inoculated (*i.n*.) with PR8 influenza virus (Figure 2A), and body weight and survival were monitored daily for nine days. *Rbm25*‐cKO mice showed greater body weight loss and increased mortality, compared with WT controls (Figure 2B,C). As depicted in Figure 2C, two *Rbm25*‐cKO mice started to die at day 5, and nine of ten mice died at day 9 post PR8 infection. In contrast, three WT mice started to die at the seventh day post PR8 infection, and seven out of ten mice survived at day 9. At 7 days post‐infection (dpi), viral titers in the lungs of *Rbm25*‐cKO mice were significantly higher compared to WT mice (Figure 2D), accompanied by elevated mRNA levels of *PR8 HA* and *IAV M1* gene (Figure 2E). IF staining revealed stronger accumulation of viral HA protein in *Rbm25*‐cKO lungs compared with WT controls (Figure 2F). In addition, the expression of viral NP protein was also substantially increased in *Rbm25*‐cKO lungs (Figure 2G). Histopathological analysis revealed that PR8‐infected *Rbm25*‐cKO mice exhibited aggravated lung tissue damage and more severe inflammatory pathology at 7 dpi, compared to their WT counterparts (Figure 2H,I). Overall, these results indicate an important role of RBM25 in protecting mice from IAV infection and in mitigating viral pathogenicity in mice.

To further assess the phenotypic consequences, WT and *Rbm25*‐cKO mice were intraperitoneally (*i.p*.) infected with HSV‐1, a neurotropic alpha herpesvirus that can infect the peripheral and central nervous systems (Figure 2J). The changes in body weight and survival were assessed for eight days. Consistent with our previous findings, we observed a similar phenomenon. Following HSV‐1 infection, the *Rbm25*‐cKO mice showed a more evident reduction in body weight than WT mice (Figure 2K). As elucidated by overall survival, *Rbm25*‐cKO mice were more susceptible to HSV‐1 infection than WT mice (Figure S4A). Concurrently, much greater titers of HSV‐1 were detected in the brain of *Rbm25*‐cKO mice, compared to WT mice (Figure 2L). Viral *HSV TK* mRNA levels were significantly higher in the brain, heart, and spleen of *Rbm25*‐cKO mice, compared to WT mice (Figure 2M). Consistently, the antibody against HSV‐1‐based immunohistochemistry (IHC) analysis indicated more HSV‐1 virions in the cerebral cortex in *Rbm25*‐cKO mice than in WT mice (Figure 2N). Hematoxylin‐eosin (H&E) staining analysis of brain tissues showed that there were more infiltrated inflammatory cells in the brains of *Rbm25*‐cKO mice than in the brains of WT mice after HSV‐1 infection (Figure 2O). Taken together, these findings indicate that RBM25 also displays defensive effects against neurotropic DNA virus infection in mice.

Similar enhanced susceptibility was also observed with VSV infection. WT and *Rbm25*‐cKO mice were *i.p*. injection with VSV, survival and body weight were monitored daily for six days (Figure S4B). *Rbm25*‐cKO mice showed increased mortality and greater body weight loss compared with WT controls (Figure S4C,D). In line with these observations, the viral titers in the liver in *Rbm25*‐cKO mice were also higher than those in WT mice (Figure S4E). The expression levels of *VSV‐G* mRNA in various tissues of *Rbm25*‐cKO mice were significantly increased comparing with WT mice (Figure S4F). H&E staining analysis showed the livers of *Rbm25*‐cKO mice exhibited severe tissue damage, characterized by ballooning degeneration of hepatocytes and inflammatory cell infiltration, and the histology score was higher than WT mice at day 1 after VSV infection (Figure S4G). Likewise, the lungs of *Rbm25*‐cKO mice revealed extensive inflammatory cell infiltration, alveolar collapse, and widespread hemorrhage and pulmonary, which were markedly attenuated in WT animals (Figure S4H). Collectively, these data demonstrate that genetic ablation of RBM25 in macrophages leads to greater susceptible to viral infections and pathogenesis in mice.

### 2.3 RBM25 is a Host Restriction Factor Against Virus Infection

**FIGURE 3:**
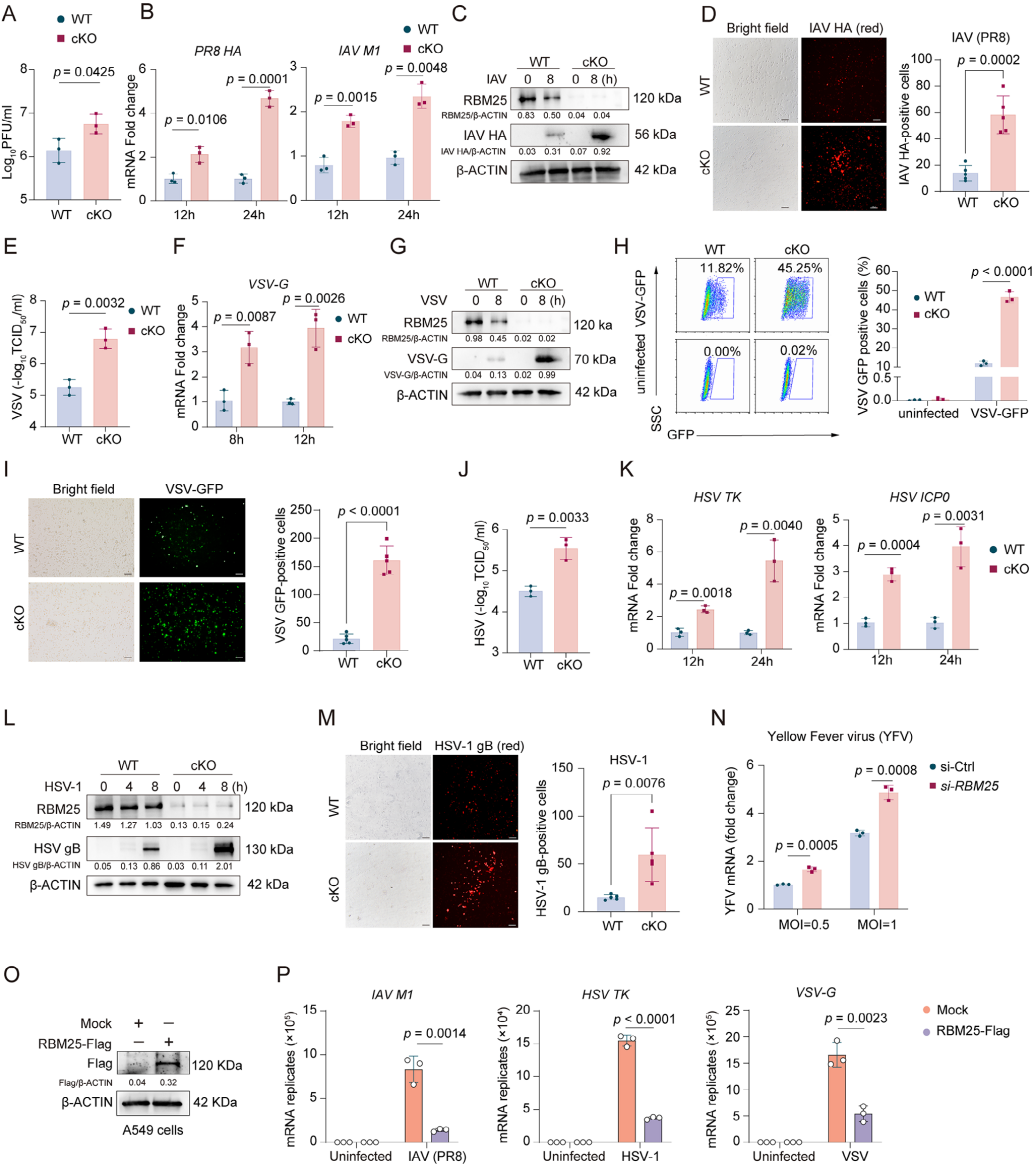
RBM25 inhibits virus infection and replication of multiple viruses in primary macrophages and human cells in vitro. (A) IAV titers in the supernatants of peritoneal macrophages from WT and *Rbm25*‐cKO mice infected with PR8 influenza virus for 24 h. (B) RT‐qPCR analysis of *PR8 HA* and *IAV M1* mRNA in peritoneal macrophages from WT and *Rbm25*‐cKO mice infected with PR8 influenza virus for 12 or 24 h. (C) Immunoblots analysis of IAV HA protein in peritoneal macrophages from WT and *Rbm25*‐cKO mice infected with PR8 influenza virus for 8 h or left untreated. (D) IF analysis of IAV HA expression in peritoneal macrophages from WT and *Rbm25*‐cKO mice (scale bar, 100 µm). The IAV HA‐positive cells were calculated by Image J software. (E) VSV titers in the supernatants of peritoneal macrophages from WT and *Rbm25*‐cKO mice infected with VSV for 12 h. (F) RT‐qPCR analysis of *VSV‐G* mRNA in peritoneal macrophages from WT and *Rbm25*‐cKO mice infected with VSV for 8 or 12 h. (G) Immunoblots analysis of VSV‐G protein of peritoneal macrophages from WT and *Rbm25*‐cKO mice infected with VSV for 8 h or left untreated. (H) Flow cytometry analysis of peritoneal macrophages from WT and *Rbm25*‐cKO mice infected with VSV‐GFP for 8 h. (I) Fluorescent microscopy imaging (left) and statistical analysis (right) of peritoneal macrophages from WT and *Rbm25*‐cKO mice infected with VSV‐GFP for 8 h (scale bar, 100 µm). The VSV‐ GFP‐positive cells were calculated by Image J software. (J) HSV‐1 titers in the supernatants of peritoneal macrophages from WT and *Rbm25*‐cKO mice infected with HSV‐1 for 24 h. (K) RT‐qPCR analysis of *HSV‐1 TK* and *ICP0* mRNA in peritoneal macrophages from WT and *Rbm25*‐cKO mice infected with HSV‐1 for 12 or 24 h. (L) Immunoblots analysis of HSV‐1 gB protein of peritoneal macrophages from WT and *Rbm25*‐cKO mice infected with HSV‐1 for 4 or 8 h. (M) IF analysis of HSV‐1 gB expression in peritoneal macrophages from WT and *Rbm25*‐cKO mice infected with HSV‐1 for 8 h (scale bar, 100 µm). The HSV‐1 gB‐positive cells were calculated by Image J software. (N) RT‐qPCR analysis of viral mRNA in Huh7 cells transfected with control siRNA (si‐Ctrl) or *RBM25* siRNA (si‐*RBM25*) followed by infection with YFV at different MOIs. (O) Immunoblot analysis of Flag‐tagged RBM25 in A549 cells transfected with RBM25‐Flag or mock plasmid for 48 h. (P) RT‐qPCR analysis of mRNA levels of *IAV M1*, *HSV TK*, and *VSV‐G* genes in A549 cells transfected with RBM25‐Flag or mock plasmid and then infected with PR8 influenza virus, HSV‐1, or VSV for the indicated times. Data are presented as the mean ± SD. Unpaired two‐tailed Student's *t*‐test (A, B, D–F, H–K, M, N, P).

To determine the impact of RBM25 on different kinds of virus infection in vitro, peritoneal macrophages from WT and *Rbm25*‐cKO mice were isolated and then infected with IAV. RBM25 deficiency significantly increased the viral titers in the supernatants at 24 h post‐infection (hpi) (Figure 3A). The mRNA levels of IAV (PR8) hemagglutinin (*PR8 HA*) and *IAV M1* gene were also significantly higher in the RBM25‐deficient peritoneal macrophages compared to the control group (Figure 3B), and the viral IAV HA protein were markedly increased (Figure 3C). IF staining and microscopy imaging assays revealed that, upon RBM25 deficiency, the replication of HA‐positive IAV were also significantly higher compared to the control group (Figure 3D). A similar effect of RBM25 genetic ablation on VSV infection was also observed, as indicated by the quantification of viral titers (Figure 3E) and viral mRNA (Figure 3F). RBM25 deficiency significantly increased VSV titers and the mRNA levels of *VSV‐G* gene. The immunoblotting results for the lysates of VSV‐infected peritoneal macrophages demonstrated that deletion of RBM25 led to increased production of viral protein VSV‐G in macrophages (Figure 3G). Consistently, the replication of VSV‐GFP (a GFP‐expressing strain of VSV) was substantially increased in the *Rbm25*‐cKO macrophages compared to the WT counterparts as monitored by flow cytometry analysis (Figure 3H) and fluorescent imaging of the GFP signals (Figure 3I), respectively.

To determine whether RBM25 also plays a role in the infection of other kinds of viruses, peritoneal macrophages from *Rbm25*‐cKO and WT mice were infected with HSV‐1 or yellow fever virus (YFV). The viral titers of HSV‐1 (Figure 3J) and the mRNA levels of viral genes *HSV‐1 TK* and *ICP0* (Figure 3K) were significantly increased in RBM25‐deficient macrophages. Moreover, the HSV‐1 gB protein level was correspondingly elevated (Figure 3L). A notable increase in gB‐positive HSV‐1 viral replication was also demonstrated by IF staining and microscopy imaging assays in RBM25‐deficient peritoneal macrophages compared to the control group (Figure 3M). Similar findings were observed in YFV, the prototypical hemorrhagic fever virus that is endemic to regions of Africa and South America, where the viral mRNA levels were significantly higher than in the control group at different MOI (Figure 3N). Furthermore, RBM25 was overexpressed in human A549 cells to assess its impact on viral replication. The mRNA levels of the *IAV M1*, *HSV‐1 TK*, and *VSV‐G* genes were markedly decreased in RBM25‐overexpressed A549 cells compared to those in mock controls (Figure 3O,P).

Next, we employed an siRNA targeting *Rbm25* to further explore the role of RBM25 in murine macrophage RAW 264.7 cell lines during infections of different kinds of viruses (Figure S5A,B). Compared to the non‐targeting siRNA control group (si‐Ctrl), siRNA‐mediated *Rbm25* silencing (si‐*Rbm25*) in RAW 264.7 cell lines led to a significant increase in VSV titers at 12 hpi (MOI = 1) (Figure S5C), as well as a marked increase in the mRNA expression levels of *VSV‐G* (Figure S5D). In *Rbm25*‐silenced RAW 264.7 cells, the VSV‐G protein expression levels were also significantly upregulated (Figure S5E). Similarly, RBM25 silencing significantly promoted HSV‐1 infection in RAW 264.7 cell lines. RBM25 silencing resulted in higher HSV‐1 viral loads than those from WT ones (Figure S5F), and the mRNA levels of viral genes *HSV‐1 TK* and *ICP0* (Figure S5G) were significantly increased. Additionally, the HSV‐1 gB protein levels were also significantly higher than that in WT group (Figure S5H). In addition, in *Rbm25*‐silenced RAW 264.7 cell lines, the mRNA levels of IAV genes such as *PR8 HA* and *IAV M1* (Figure S5I) and HA protein expression (Figure S5J) were significantly elevated compared with the control group. In summary, RBM25 inhibits the replication of various kinds of pathogenic RNA and DNA viruses in vitro.

### 2.4 RBM25 Mediates Antiviral Protection Independent of IFN‐I Signaling Pathway

**FIGURE 4:**
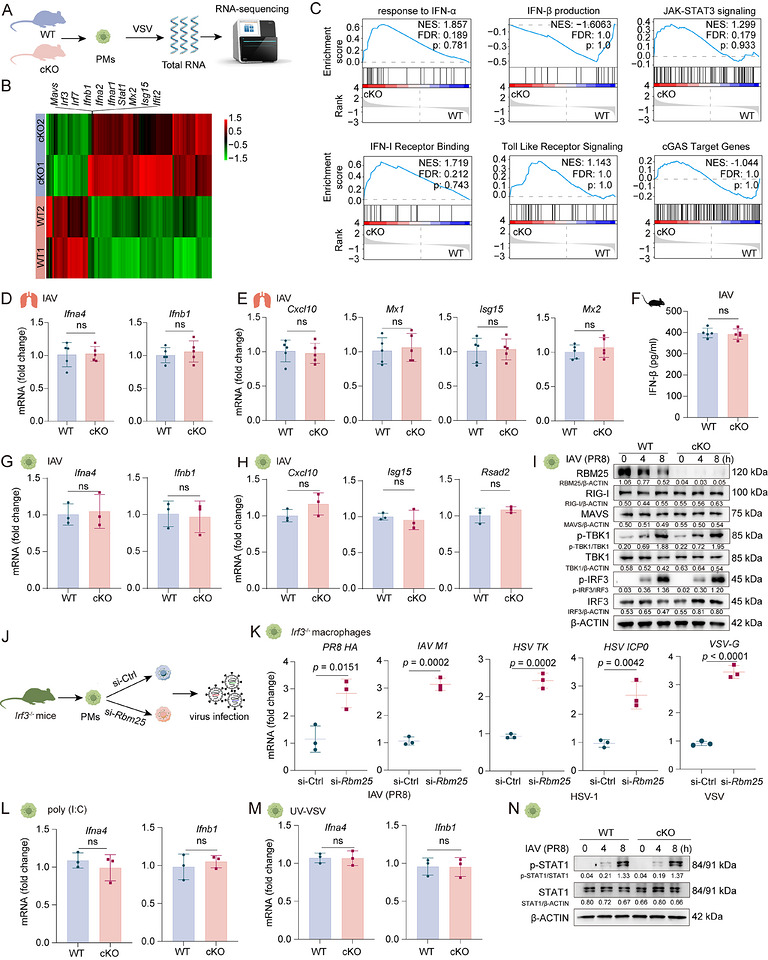
RBM25 mediates antiviral protection independent of the IFN‐I signaling pathway. (A) A scheme of RNA‐seq analysis of WT and *Rbm25*‐cKO peritoneal macrophages infected with VSV for 4 h. (B) Heatmap of differentially expressed genes (DEGs) in *Rbm25*‐deficient and WT peritoneal macrophages infected with VSV for 4 h. (C) GSEA showing enrichment of DEGs in the pathways involving in the response to IFN‐α, IFN‐β production, JAK‐STAT3, IFN‐I receptor binding, toll like receptor, and cGAS target gene signaling pathways. (D) RT‐qPCR analysis of IFN‐I (*Ifna4, Ifnb1*) mRNA in the lung tissues from WT and *Rbm25*‐cKO mice infected with PR8 influenza virus with a sublethal dose (50 PFU., *n* = 5 per group) at 7 dpi. (E) RT‐qPCR analysis of *Cxcl10, Mx1, Isg15*, and *Mx2* mRNA in the lung tissues from WT and *Rbm25*‐cKO mice treated as in (D). (F) ELISA of IFN‐β in serum from *Rbm25*‐cKO and WT mice infected with PR8 influenza virus, as in (D). (G) RT‐qPCR analysis of IFN‐I (*Ifna4, Ifnb1*) mRNA in peritoneal macrophages from *Rbm25*‐cKO and WT mice infected with PR8 influenza virus for 12 h. (H) RT‐qPCR analysis of *Cxcl10, Isg15*, and *Rsad2* mRNA in peritoneal macrophages from WT and *Rbm25*‐cKO mice treated as in (G). (I) Immunoblots analysis of RBM25, RIG‐I, MAVS, p‐TBK1, TBK1, p‐IRF3, and IRF3 in peritoneal macrophages from WT and *Rbm25*‐cKO mice infected with PR8 influenza virus for the indicated times. β‐ACTIN was used as a loading control. (J) A scheme of experiment analyzing the infection of viruses in peritoneal macrophages from *Irf3* ^−/−^ mice transfected with control siRNA or siRNA targeting *Rbm25*. (K) RT‐qPCR analysis of *PR8 HA* and *IAV M1, HSV‐1 TK* and *ICP0, VSV‐G* mRNA in peritoneal macrophages from *Irf3* ^−/−^ mice transfected with control siRNA or siRNA targeting *Rbm25* followed by infection with PR8 influenza virus for 12 h, HSV‐1 for 12 h, or VSV for 8 h, respectively. (L, M) RT‐qPCR analysis of IFN‐I (*Ifna4, Ifnb1*) mRNA in *Rbm25*‐cKO and WT peritoneal macrophages transfected with poly (I:C) for 4 h (L) or stimulated with UV‐VSV for 18 h (M). (N) Immunoblots analysis of p‐STAT1 and STAT1 in peritoneal macrophages from WT and *Rbm25*‐cKO mice infected with PR8 influenza virus for the indicated times. β‐ACTIN was used as a loading control. Data are presented as the mean ± SD. Unpaired two‐tailed Student's *t*‐test (D–H, K–M). ns, not significant.

Considering the pivotal role of IFN‐I signaling in antiviral immunity, we next sought to determine whether RBM25 modulates this canonical antiviral TBK1‐IRF3 pathway and IFN‐I production. To address this, peritoneal macrophages were isolated from *Rbm25*‐cKO and WT mice, followed by infection with VSV for 4 h, and RNA sequencing (RNA‐seq) was performed (Figure 4A). The heatmap shows no significant difference between RBM25‐deficient macrophages compared to controls in the expression of key genes involved in the IFN‐I pathway, including signaling transduction molecules (*Mavs*, *Irf3*, and *Irf7*), IFN‐I genes (*Ifna2, Ifnb1*), components of the Janus kinase‐signal transducer and activator of transcription (JAK‐STAT) pathway, or several typical interferon‐stimulated genes (ISGs) such as *Isg15, Ifit2*, and *Mx2* (Figure 4B). Gene set enrichment analysis (GSEA) analysis also revealed no significant change in IFN‐I signaling pathways, including response to IFN‐α, IFN‐β production, JAK‐STAT3, IFN‐I receptor binding, Toll‐like receptor, and cGAS target gene signaling pathways (Figure 4C). These data together suggested that RBM25 might have no influence on the IFN‐I production and effects.

To further investigate this guess, we performed in vivo and in vitro infection studies with PR8 influenza virus, HSV‐1 or VSV. RT‐qPCR analysis of lung homogenates revealed no significant differences in the mRNA levels of IFN‐I (*Ifna4, Ifnb1*) (Figure 4D) or representative ISGs (*Cxcl10, Mx1, Isg15*, and *Mx2*) (Figure 4E) between WT and *Rbm25*‐cKO mice. These findings were confirmed by comparable serum concentrations of IFN‐β in both *Rbm25*‐cKO and WT mice following IAV infection (Figure 4F). In vitro, peritoneal macrophages from *Rbm25*‐cKO mice infected with IAV, or HSV‐1 also exhibited no significant changes in IFN‐I or ISG mRNA expression relative to WT controls (Figure 4G,H and Figure S6A,B). We also detected no measurable difference in IFN‐β secretion in the supernatants of peritoneal macrophages from *Rbm25*‐cKO and WT mice after HSV‐1 infection (Figure S6C). Consistent with this observation, VSV infection likewise failed to elicit a differential IFN‐β response between genotypes (Figure S6D–F).

To explore the molecular mechanisms underlying these observations, we assessed the activation status of key signaling transduction components in the TBK1‐IRF3 pathway following IAV infection by immunoblotting. The results indicated that RBM25 deficiency did not affect the protein levels of RIG‐I or MAVS, which are critical for the RNA virus‐mediated IFN‐I signaling pathway. Additionally, the phosphorylation and activation of TBK1 and IRF3 were also comparable between RBM25‐deficient and control groups. These findings collectively suggest that the antiviral effects of RBM25 is independent of the regulation of the canonical TBK1‐IRF3 signaling pathway (Figure 4I and Figure S6G). To further validate this possibility, we employed IRF3 knockout (*Irf3* ^−/−^) mice [28], the model lacking an effective IFN‐I system, to investigate the role of IFN‐I in RBM25‐mediated host defense. Peritoneal macrophages isolated from *Irf3* ^−/−^ mice were infected with IAV, HSV‐1, or VSV, respectively. Notably, RBM25‐deficient macrophages still exhibited significantly higher levels of viral gene expression compared to control cells (Figure 4K), reinforcing the conclusion that the antiviral activity of RBM25 is independent of modulation of TBK1‐IRF3 pathway‐dependent IFN‐I production.

Furthermore, peritoneal macrophages from *Rbm25*‐cKO and WT mice were subjected to the RNA viral mimic poly (I:C) transfection. No significant differences were observed in the mRNA expression of IFN‐I (Figure 4L) or representative ISGs (*Isg15, Cxcl10, Mx1, Rsad2*, and *Ifit2*) (Figure S6H). In addition, upon stimulation with UV‐VSV, no significant differences were observed in IFN‐I and ISG production between peritoneal macrophages from *Rbm25*‐cKO and WT mice (Figure 4M and Figure S6I). Immunoblot analysis further demonstrated that the phosphorylation and subsequent activation of STAT1, a key downstream effector of the TBK1‐IRF3 pathway, were also unaffected (Figure 4N). Similarly, direct stimulation with IFN‐β protein also failed to elicit any differential ISG mRNA expression in *Rbm25*‐cKO peritoneal macrophages compared to WT controls (Figure S6J).

### 2.5 RBM25 Restricts Virus Penetration in Both Human and Mouse Cells

**FIGURE 5:**
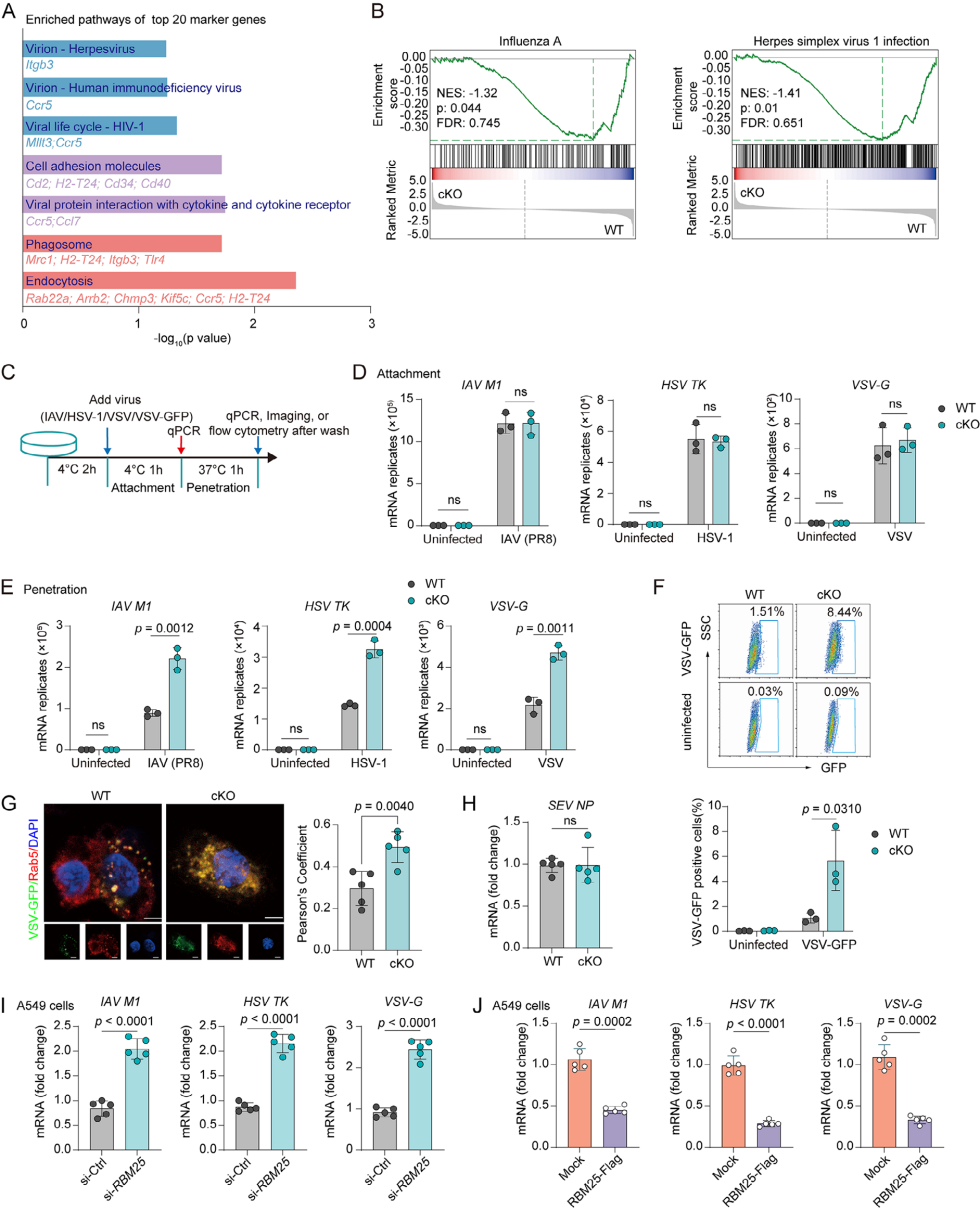
RBM25 specifically restricts virus penetration in both human and mouse cells. (A) KEGG enrichment analysis of DEGs in WT and *Rbm25*‐cKO peritoneal macrophages infected with VSV for 4 h. (B) GSEA analysis of the pathway involving in the response to influenza A and HSV‐1 pathways. (C) A scheme of experiments analyzing the attachment and the penetration of viral infection. (D) RT‐qPCR analysis of *IAV M1*, *HSV TK*, and *VSV‐G* mRNA of attached viruses in peritoneal macrophages from WT and *Rbm25*‐cKO mice treated as in (C). (E) RT‐qPCR analysis of *IAV M1*, *HSV TK*, and *VSV‐G* mRNA of the penetrated viruses in peritoneal macrophages from WT and *Rbm25*‐cKO mice treated as in (C). (F) Flow cytometry analysis of the penetrated viruses in peritoneal macrophages from WT and *Rbm25*‐cKO mice treated with VSV‐GFP as in (C). (G) IF confocal analysis of Rab5 and VSV‐GFP in peritoneal macrophages from WT and *Rbm25*‐cKO mice treated with VSV‐GFP as in (C) (scale bar, 5 µm). Pearson's Coefficient was calculated by the Image J software. (H) RT‐qPCR analysis of *SEV NP* mRNA of Sendai virus in peritoneal macrophages from WT and *Rbm25*‐cKO mice for 12 h. (I) RT‐qPCR analysis of *IAV M1*, *HSV TK*, and *VSV‐G* mRNA levels of the corresponding penetrated viruses in A549 cells transfected with si‐Ctrl or si‐*RBM25*. (J) RT‐qPCR analysis of *IAV M1*, *HSV TK*, and *VSV‐G* mRNA levels of the corresponding penetrated viruses in A549 cells transfected with RBM25‐Flag or mock plasmid. Data are presented as the mean ± SD. Unpaired two‐tailed Student's *t*‐test (D–J). ns, not significant.

We identified and conducted pathway enrichment analysis. KEGG pathway analysis from RNA‐seq data revealed that the DEGs were predominantly enriched in viral entry‐related pathways, such as the endocytosis and phagosome pathways (Figure 5A). In addition, significant enrichment was also observed in pathways associated with viral infection (e.g., virion‐herpesvirus and viral life cycle) and the cell adhesion molecules pathway. Consistent with KEGG enrichment results, GSEA analysis also revealed a significant enrichment of the viral infection‐related gene set (e.g., influenza A and herpesvirus infection) in the experimental group (Figure 5B). These data suggested that RBM25 may modulate the expression of genes associated with the viral infection process.

Viral infection is a multistep process, including the early entry and replication stage and the late assembly and release stage. We next examined whether knockout of RBM25 affected the early infection process. Peritoneal macrophages from *Rbm25*‐cKO and WT mice were precooled at 4°C for 2 h and then infected with three viruses, including IAV, HSV‐1, or VSV, respectively at 4°C for 1 h to allow the virus to attach to the cells. The cells were washed with cold PBS and lysed to prepare viral genomic RNA, following by RT‐qPCR analysis. Alternatively, the cells were incubated at 37°C for 1 h to allow IAV, HSV‐1, or VSV penetration into cells. The cells were then washed with cold PBS followed by viral genomic RNA preparation, immunofluorescent staining, and imaging analysis or flow cytometry analysis (Figure 5C). Results from RT‐qPCR analysis suggested that the mRNA levels of *IAV M1* gene, *HSV T*K gene, or *VSV‐G* gene of the attached viruses IAV, HSV‐1, or VSV were comparable between the peritoneal macrophages from *Rbm25*‐cKO and WT mice (Figure 5D). Whereas the levels of *IAV M1* gene, *HSV TK* gene, or *VSV‐G* gene of penetrated viruses IAV, HSV‐1, or VSV were significantly higher in *Rbm25*‐cKO macrophages than in WT ones (Figure 5E). These data together suggest that knockout of RBM25 increased the penetration of viruses into but not the attachment of viruses to peritoneal macrophages. Consistently, the penetration of VSV‐GFP was substantially increased in the *Rbm25*‐cKO macrophages compared to the WT counterparts as monitored by flow cytometry analysis (Figure 5F).

Many viruses enter host cells via endocytosis, where Rab5‐mediated maturation of early endosomes into acidic late compartments triggers critical conformational changes in the virion, enabling endosomal escape and cytoplasmic release of the viral genome. Consequently, co‐localization of viral particles with Rab5 serves as a definitive marker for successful entry through this canonical pathway [29, 30, 31]. To investigate the role of RBM25 in this process, peritoneal macrophages from *Rbm25*‐cKO and WT mice were infected with VSV‐GFP for 1 h, following by IF staining for Rab5. Confocal imaging analysis revealed the co‐localization of VSV‐GFP and Rab5 in *Rbm25*‐cKO macrophages was significantly higher compared to WT controls (Figure 5G), indicating that RBM25 deficiency enhances the early endosomal entry of VSV, suggesting an enhanced rate of viral internalization. Furthermore, we observed whether RBM25 affected the infection of Sendai virus (SEV), which enters host cells exclusively via membrane fusion rather than the endocytic pathway. The results showed that the mRNA level of SEV NP gene exhibited no significant difference between *Rbm25*‐cKO macrophages and WT control groups (Figure 5H). Collectively, these data indicate that RBM25 restricts the penetration of a broad spectrum of DNA and RNA viruses into macrophages.

To further confirm the role of RBM25 in viral entry, viral entry assays were performed in human A549 cells with RBM25 knockdown or overexpression. RBM25 knockdown significantly increased the mRNA levels of penetrated viral genes (*IAV M1*, *HSV‐1 TK*, and *VSV‐G*) in A549 cells (Figure 5I and Figure S7). Correspondingly, RBM25 overexpression inhibited mRNA levels of the above penetrated viral genes (Figure 5J). These data confirm that RBM25 inhibits virus infection by impeding virus penetration in human and mouse cells.

### 2.6 RBM25 Inhibits Rab22a Protein Expression to Restrict Viral Entry

**FIGURE 6:**
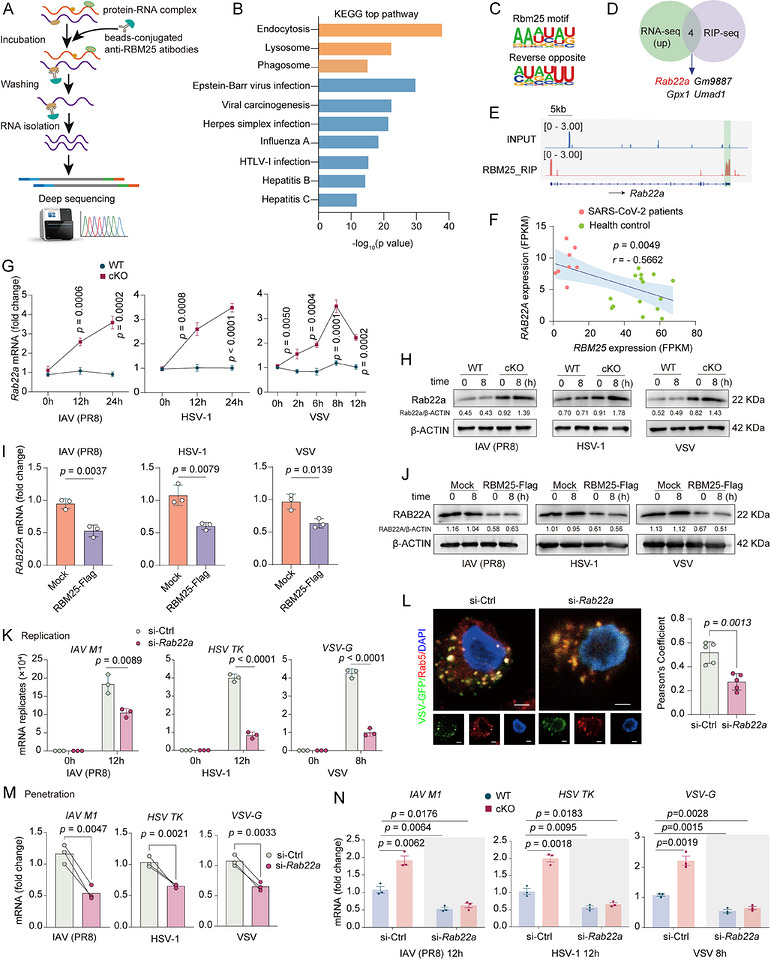
RBM25 inhibits Rab22a protein expression to restrict viral entry. (A) Schematic of the experimental workflow for RIP‐seq using antibody against RBM25 in bone‐marrow‐derived macrophages. (B) KEGG enrichment analysis of RBM25‐binding RNAs identified by RIP‐seq. (C) RBM25 motif and reverse opposite motif in RIP‐seq by HOMER. (D) Venn diagram analysis showed the overlap between up‐regulated genes in RNA‐seq data and RBM25‐bound genes identified by RIP‐seq. (E) IGV analysis of RBM25‐binding peaks at the gene locus of *Rab22a* in WT BMDMs by RIP analysis. (F) Correlation analysis between *RBM25* and *RAB22A* expression in PBMCs from patients infected with SARS‐CoV‐2 and health control (GSE215262, Spearman correlation analysis). (G, H) RT‐qPCR analysis of *Rab22a* mRNA levels (G) and immunoblot analysis of Rab22a protein (H) in WT and *Rbm25*‐cKO peritoneal macrophages infected with PR8 influenza virus, HSV‐1, or VSV for the indicated times. (I, J) RT‐qPCR analysis (I) and immunoblot analysis (J) of *RAB22A* in A549 cells transfected with RBM25‐Flag or mock plasmid and then infected with PR8 influenza virus, HSV‐1, or VSV for the indicated times. (K) RT‐qPCR analysis of *IAV M1*, *HSV TK*, and *VSV‐G* mRNA in peritoneal macrophages transfected with si‐Ctrl or si‐*Rab22a* followed by infected with PR8 influenza virus, HSV‐1, or VSV for the indicated times. (L) IF confocal analysis of Rab5 and VSV‐GFP in peritoneal macrophages transfected with si‐Ctrl or si‐*Rab22a* followed by treated with VSV‐GFP to detect the penetrated viruses (scale bar, 5 µm). (M) RT‐qPCR analysis of the viral mRNA in the penetrated viruses in peritoneal macrophages transfected with si‐Ctrl or si‐*Rab22a*. (N) RT‐qPCR analysis of viral mRNA in WT and *Rbm25*‐cKO peritoneal macrophages transfected with si‐Ctrl or si‐*Rab22*a followed by infection with PR8 influenza virus, HSV‐1, or VSV for the indicated times. Data are presented as the mean ± SD. Unpaired two‐tailed Student's *t*‐test (G, I, K–M), two‐way ANOVA test (N).

As an RBP, we suspected whether RBM25 may exert its antiviral effects in direct RNA binding‐dependent manners. To elucidate the potential mechanisms underlying RBM25‐mediated restriction of viral entry, we performed endogenous RNA immunoprecipitation followed by high‐throughput sequencing (RIP‐seq) using an antibody against RBM25 to identify target RNAs that physically interact with RBM25 in macrophages (Figure 6A). KEGG analysis of the RIP‐seq data revealed substantial enrichment in endocytic and phagosomal pathways, including endocytosis, lysosome, and phagosome. Additionally, multiple viral infection‐associated pathways were highly enriched, such as Epstein‐Barr virus infection, herpes simplex infection, and influenza A virus infection (Figure 6B). Our RIP‐seq analysis identified a conserved “AAAUAU” motif within the RBM25‐bound RNAs (Figure 6C). These findings indicated that RBM25, as an RBP, may specifically bind to RNAs associated with viral endocytosis and thereby modulate the viral infection process. Subsequently, we performed a Venn intersection analysis between the upregulated genes from the RNA‐seq and the genes identified in our RBM25 RIP‐seq dataset. Among them, *Rab22a*, which encodes the host GTPase Rab22a, a protein implicated in membrane trafficking and intracellular transport‐attracting our particular attention (Figure 6D). As a member of the RAB family of small GTPases, Rab22a exerts a pivotal role in the regulation of intracellular membrane trafficking, and is specifically characterized by its functional involvement in the mediation of endosomal transport and sorting processes [32]. Integrative Genomics Viewer (IGV) visualization further confirmed a prominent binding peak of RBM25 in the 3' UTR of *Rab22a* mRNA (Figure 6E), indicating that RBM25 may regulate Rab22a expression through direct binding to its mRNA. Additionally, a significant negative correlation between *RBM25* and *RAB22A* mRNA expression was found in PBMCs from SARS‐CoV‐2‐infected population and health control (Figure 6F). Given the crucial function of endosomes in processing and sorting intracellular cargo, we hypothesized that Rab22a may mediate the restrictive effect of RBM25 on viral infection.

We next investigated whether Rab22a expression is altered upon viral infection in *Rbm25*‐cKO macrophages. Following infection with IAV, HSV‐1, or VSV, RT‐qPCR analysis revealed a marked increase in *Rab22a* mRNA levels in RBM25‐deficient macrophages, whereas viral stimulation induced negligible changes of *Rab22a* mRNA in WT controls (Figure 6G). Consistent with these findings, immunoblot analysis demonstrated that protein levels of Rab22a were also significantly elevated in *Rbm25*‐cKO macrophages infected with IAV, HSV‐1, or VSV (Figure 6H). In contrast, the mRNA and protein levels of RAB22A were significantly reduced in RBM25‐overexpressed A549 cells compared with control cells (Figure 6I,J). Analysis of our RNA‐seq data revealed that the expression of other Rab genes associated with viral infection remained unchanged upon RBM25 deficiency (Figure S8A). These results suggest that RBM25 exerts its antiviral function by modulating the expression of the endosomal trafficking regulator Rab22a.

Given prior evidence that Rab22a cooperates with Rab5 and NS4B to facilitate classical swine fever virus entry [33], we sought to determine whether Rab22a upregulation also contributes to the viral entry process under investigation. Thus, we further investigate whether Rab22a also participates in the replication, penetration, or attachment processes of IAV, HSV‐1, and VSV by siRNA‐mediated silencing Rab22a expression. Three siRNA pairs targeting *Rab22a* were designed and synthesized for in vitro RNA interference experiments. Efficient depletion of Rab22a was confirmed at both the mRNA and protein levels by RT‐qPCR and immunoblot analysis, respectively (Figure S8B,C). The siRNA demonstrating the highest silencing efficiency (si‐*Rab22a* #2) was selected for subsequent investigations. To evaluate the impact of Rab22a depletion on viral replication, the mRNA levels of the *IAV M1*, *HSV TK*, and *VSV‐G* genes were determined by RT‐qPCR assays. A significant reduction in the expression of these viral genes was observed following RNAi of *Rab22a* (Figure 6K), indicating impaired replication of the virus.

The antiviral effect of Rab22a RNAi on viral entry was next evaluated using a VSV‐GFP model. A marked reduction in VSV‐GFP entry was observed upon Rab22a silencing by IF analysis (Figure 6L). Consistent with this, RT‐qPCR analysis detected a significant decrease in the mRNA levels of the penetrated *IAV M1*, *HSV TK*, and *VSV‐G* genes following *Rab22a* silencing (Figure 6M), although the adsorption levels of the three viruses showed no significant difference (Figure S8D), further confirming the attenuation of viral entry. Based on the critical role of Rab22a in the entry of IAV, HSV‐1, and VSV, *Rab22a* was subsequently silenced in either *RBM25*‐cKO or WT macrophages to investigate its functional relationship with RBM25 (Figure S8E). The increased mRNA levels of the *IAV M1*, *HSV TK*, and *VSV‐G* genes induced by RBM25 deficiency were found to be effectively reversed following *Rab22a* silencing, as determined by RT‐qPCR analysis (Figure 6N), suggesting the enhanced viral entry observed in RBM25‐deficient macrophages is mediated by the upregulation of Rab22a protein.

### 2.7 RBM25 Interacts With RC3H1 to Destabilize *Rab22a* mRNA

**FIGURE 7:**
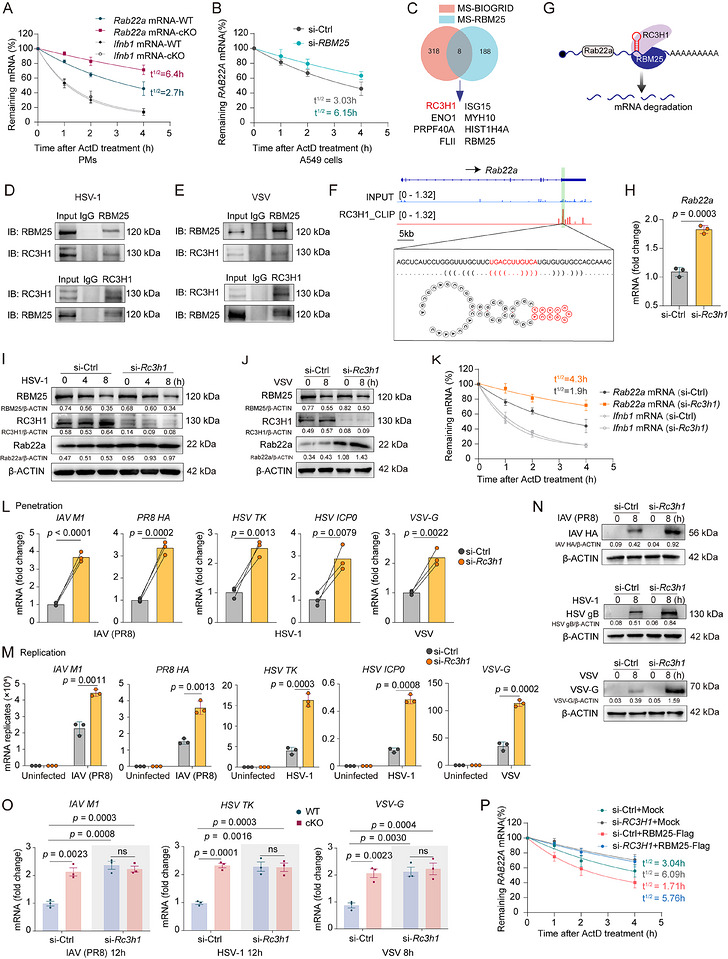
RBM25 interacts with RC3H1 to selectively reduce *Rab22a* mRNA stability. (A) The stability of *Rab22a* mRNA in WT and *Rbm25*‐cKO peritoneal macrophages infected with VSV for 4 h and then treated with 5 µg/mL actinomycin D. *Rab22a* mRNA levels were quantified by RT‐qPCR, *Ifnb1* mRNA was used as a control. (B) The stability of *RAB22A* mRNA stability in A549 cells transfected with si‐Ctrl or si‐*RBM25*. Cells were infected with VSV for 4 h and then treated with actinomycin D. *RAB22A* mRNA levels were quantified by RT‐qPCR. (C) Venn diagram of RBM25‐bound proteins detected by mass spectrometry (MS‐RBM25) and RBM25‐bound proteins predicted with BioGRID database (MS‐BIOGRID). (D, E) Immunoprecipitation (IP) analysis of the interaction between RBM25 and RC3H1 in peritoneal macrophages treated with HSV‐1 (D) or VSV (E) for 4 h. (F) IGV analysis showed RC3H1 bound to the 3'‐UTR of *Rab22a* mRNA in CLIP‐seq data (GSE132081), INPUT, GSM3842252; RC3H1_CLIP, GSM3842253. (G) Working model of the interaction between RBM25 and RC3H1 co‐binding at the 3'UTR region of *Rab22a* mRNA. (H) RT‐qPCR analysis of *Rab22a* mRNA in WT peritoneal macrophages transfected with si‐Ctrl or si‐*Rc3h1* for 48 h. (I, J) Immunoblot analysis of RBM25, RC3H1, and Rab22a protein in WT peritoneal macrophages transfected with si‐Ctrl or si‐*Rc3h1* and then infected with HSV‐1 (I) or VSV (J) for the indicated times. (K) RT‐qPCR analysis of the stability of *Rab22a* mRNA in WT peritoneal macrophages transfected with si‐Ctrl or si‐*Rc3h1* and then treated with 5 µg/mL actinomycin D. (L) RT‐qPCR analysis of *IAV M1*, *PR8 HA*, *HSV TK*, *ICP0*, and *VSV‐G* mRNA of the penetrated viruses in peritoneal macrophages transfected with si‐Ctrl or si‐*Rc3h1*. (M) RT‐qPCR analysis of *IAV M1*, *PR8 HA*, *HSV TK*, *ICP0*, and *VSV‐G* mRNA in peritoneal macrophages transfected with si‐Ctrl or si‐*Rc3h1* followed by infection with PR8 influenza virus for 12 h, HSV‐1 for 12 h, or VSV for 8 h. (N) Immunoblot analysis of IAV HA protein, HSV gB protein, and VSV‐G protein in WT peritoneal macrophages transfected with si‐Ctrl or si‐*Rc3h1* and then infected with indicated viruses. (O) RT‐qPCR analysis of *IAV M1*, *HSV TK*, and *VSV‐G* mRNA of WT and *Rbm25*‐cKO peritoneal macrophages transfected with si‐Ctrl or si‐*Rc3h1* followed by infection with PR8 influenza virus for 12 h, HSV‐1 for 12 h, or VSV for 8 h. (P) The stability of *RAB22A* mRNA stability in A549 cells transfected with si‐Ctrl or si‐*RC3H1*, along with RBM25‐Flag or mock plasmid for 48 h. Cells were infected with VSV for 4 h and then treated with 5 µg/mL actinomycin D. Data are presented as the mean ± SD. Unpaired two‐tailed Student's *t*‐test (H, L, M), two‐way ANOVA test (O). ns, not significant.

Our results demonstrated that both the mRNA and protein expression levels of Rab22a were markedly upregulated following RBM25 depletion. We therefore hypothesized that RBM25 modulates the stability of *Rab22a* mRNA. To test this hypothesis, an mRNA stability assay was performed. Actinomycin D (ActD) chase experiments revealed that RBM25 depletion significantly delayed the degradation of *Rab22a* mRNA, whereas it exerted no discernible effect on the mRNA decay of the control gene *Ifnb1* (Figure 7A). Consistently, the decreased degradation of *RAB22A* mRNA was also observed in RBM25‐silenced human A549 cells (Figure 7B). To further elucidate the underlying regulatory mechanism of RBM25, an investigation was next conducted to determine whether RBM25 regulates *Rab22a* mRNA stability by interacting with other RBPs (Figure 7C). Among these candidates, RC3H1 (also designated as Roquin1) is a well‐characterized RBP that directly controls mRNA homeostasis via binding to specific structural elements within the 3'UTRs of target mRNAs [34]. To validate the Co‐IP/MS findings, endogenous co‐immunoprecipitation assays were conducted using an anti‐RBM25‐specific antibody in mouse macrophages. The results showed that RBM25 physically interacts with RC3H1 in HSV‐1‐infected macrophages. Reciprocal co‐immunoprecipitation assays using an anti‐RC3H1 antibody further verified the endogenous association between RC3H1 and RBM25 (Figure 7D). Additionally, similar results were observed in VSV‐infected mouse peritoneal macrophages (Figure 7E). Interestingly, RC3H1 mRNA and protein levels remained unaltered following viral infection (Figure S9A,B).

Functionally, RC3H1 exerts post‐transcriptional regulation by specifically specific structural motifs in mRNA 3’UTRs, thereby recruiting the deadenylate complex to promote mRNA decay and facilitating K48‐linked ubiquitination of target proteins. Through these dual mechanisms, it downregulates key factors involved in immune, inflammatory, and stress‐response pathways [35, 36]. However, whether RC3H1 modulates the mRNA stability of *Rab22a* to control viral entry during viral infection remains unclear. We identified RC3H1 target mRNAs through targeted database mining of published data [37], followed by IGV visualization and secondary structure prediction. A prominent RC3H1 binding peak was identified at an AU‐rich stem‐loop in the Rab22a 3'UTR (Figure 7F), suggesting that RC3H1 regulates Rab22a mRNA stability via direct binding to this conserved motif. Based on the finding, we hypothesize that RBM25 recruits RC3H1 to cooperatively act on the 3′UTR of *Rab22a* mRNA, thereby reducing its stability (Figure 7G). To further verify the hypothesis, *Rc3h1* gene expression was silenced in mouse peritoneal macrophages. Efficient RNAi of RC3H1 was confirmed at the mRNA level by and at the protein level, respectively (Figure S9C,D). The siRNA with the most potent silencing efficiency (si‐*Rc3h1* #3) was selected for subsequent experiments, and RT‐qPCR analysis revealed a significant upregulation of *Rab22a* mRNA expression in *Rc3h1*‐silenced macrophages (Figure 7H). Immunoblot analysis further confirmed that protein levels of Rab22a were also markedly increased following *Rc3h1* silencing in both HSV‐1‐infected (Figure 7I) and VSV‐infected (Figure 7J) macrophages. Subsequently, an mRNA stability assay was conducted. ActD chase experiments demonstrated that *Rc3h1* silencing significantly delayed *Rab22a* mRNA degradation, whereas no discernible effect was exerted on the mRNA decay of the control gene *Ifnb1* (Figure 7K).

Subsequently, we silenced *Rc3h1* expression in mouse peritoneal macrophages and examined its effect on the viral entry of IAV, HSV‐1, and VSV. The results showed that the mRNA levels of the *IAV M1* and *PR8 HA* genes, the *HSV TK* and *ICP0* genes, and the *VSV‐G* gene of the penetrated viruses were significantly higher in *Rc3h1*‐silenced cells compared to control cells (Figure 7L), suggesting that RC3H1 restricts the entry of the virus. Furthermore, we assessed the replication of these viruses following *R3ch1* silencing in mouse macrophages. RT‐qPCR results also demonstrated that the mRNA levels of the *IAV M1* and *PR8 HA* genes, *HSV TK* and *ICP0* genes, and *VSV‐G* gene were significantly increased upon *Rc3h1* silencing (Figure 7M). Similarly, the expression levels of IAV HA protein, HSV‐1 gB protein, and VSV‐G protein were markedly elevated in *R3ch1‐*silenced macrophages, compared to controls (Figure 7N), indicating RC3H1 deficiency enhances viral infection by promoting viral entry. Finally, we silenced *Rc3h1* expression in macrophages from both *Rbm25*‐cKO and WT mice and infected them with the indicated viruses to observe the influences on viral replication (Figure S9E). The results revealed that the mRNA levels of the *IAV M1* gene, *HSV TK* gene, *VSV‐G* gene, and *Rab22a* gene were significantly elevated upon RNAi‐mediated silencing of *Rc3h1* or depletion of *Rbm25*. However, silencing of *Rc3h1* in RBM25‐deficient macrophages did not cause a further significant increase in the levels of these above genes (Figure 7O and Figure S9F). Furthermore, ActD chase experiments showed that RC3H1 silencing alone delayed *RAB22A* mRNA degradation in A549 cells, while overexpression of RBM25 failed to decrease *RAB22A* mRNA stability in RC3H1‐silenced A549 cells (Figure 7P).

Collectively, our study identifies RBM25 as a broad‐spectrum host antiviral factor that restricts virus endocytosis and entry by recruiting RC3H1 to destabilize the mRNA encoding pro‐entry factor Rab22a (Figure S10).

## 3 Discussion

Viral infection‐induced downregulation of host restriction factors is a common strategy employed by viruses to evade host immunity. Consistent with this notion, our results demonstrated decreased RBM25 expression following viral infection, implying that viruses may target RBM25 to promote their replication. Supporting this hypothesis, RBM25 deficiency in mice led to heightened susceptibility to multiple viral infections and exacerbated tissue damage, while in vitro experiments confirmed that RBM25 inhibits the infection and replication of diverse viruses. These findings collectively establish RBM25 as an intrinsic host restriction factor with broad antiviral activity, distinguishing it from many IFN‐dependent factors that exhibit limited antiviral spectra or require pre‐activation. Notably, unlike canonical antiviral factors such as PKR or OAS, the antiviral function of RBM25 is not restricted to a specific viral family, highlighting its potential as a target for broad‐spectrum antiviral intervention.

A key insight from our study is that RBM25 mediates antiviral protection independently of the IFN‐I signaling pathway. This is particularly significant given the widespread viral strategies to evade the IFN response, making IFN‐independent restriction mechanisms attractive targets for therapeutic development. Mechanistic studies demonstrated that RBM25 exerts its antiviral effect by targeting an early step in the viral life cycle: host cell entry. RBM25 deficiency significantly increased viral penetration, as evidenced by elevated mRNA levels of viral gene in penetrated viruses. This early blockade distinguishes RBM25 from typical host antiviral factors that target later stages. Its identification expands the repertoire of host factors known to directly interfere with viral entry.

To delineate the molecular basis of RBM25 antiviral activity, we identified Rab22a as a critical downstream mediator. Loss of RBM25 led to upregulation of Rab22a, a host GTPase known to facilitate viral endocytosis. This upregulation of Rab22a, in turn, potentiated viral entry, establishing a direct link between RBM25 deficiency and enhanced viral infection. Importantly, we further uncovered that RBM25 exerts its regulatory effect on Rab22a through a post‐transcriptional mechanism: RBM25 interacts with RC3H1, a well‐characterized RNA‐binding protein that modulates mRNA stability via binding to 3’UTR stem‐loop motifs. The RBM25/RC3H1 complex specifically binds to the 3’UTR of Rab22a mRNA, leading to its destabilization. This finding is particularly novel, as RC3H1 has previously been implicated in regulating T cell fate and cytokine production, but its role in antiviral immunity and interaction with RBM25 had not been reported. Thus, we identify a novel post‐transcriptional regulatory axis for Rab22a, mediated by an RNA‐binding protein complex during viral infection.

Despite these insights, several limitations of our study warrant consideration. We focused on Rab22a as the downstream target of RBM25, but it remains unclear whether RBM25 regulates other Rab GTPases involved in viral entry or additional host factors. In addition, the precise molecular details of how Rab22a facilitates viral entry (e.g., interaction with viral envelope proteins, modulation of endocytic trafficking) and how the RBM25/RC3H1 complex recognizes the 3’UTR of *Rab22a* mRNA require further investigation. Future research directions should address these limitations, including verifying the RBM25/RC3H1‐Rab22a axis in human systems, identifying the specific binding motifs within *Rab22a* mRNA 3’UTR that mediate interaction with the RBM25/RC3H1 complex, and exploring whether viruses encode proteins that antagonize RBM25 function. Additionally, preclinical studies evaluating the therapeutic potential of targeting this axis (e.g., overexpression of RBM25, inhibition of Rab22a) are warranted.

Collectively, our study unveils the RBM25/RC3H1‐Rab22a axis as a fundamental component of intrinsic immunity that restricts viral entry through mRNA stability regulation. This pathway represents a significant advance in our understanding of IFN‐independent antiviral mechanisms, providing a new framework for exploring host‐virus interactions. From a translational perspective, the broad‐spectrum activity of RBM25 suggests that targeting the RBM25/RC3H1‐Rab22a axis could lead to the development of novel antiviral strategies effective against diverse viral pathogens, including those that evade the IFN‐I response.

## 4 Experimental Section

### 4.1 Mice

*Rbm25*‐flox mice (*Rbm25* ^flox/flox^) was generated and kept in our lab, as described previously [27]. B6.129P2‐*Lyz2* ^1(cre)Ifo^/J (Strain No: 004781) mice were from the Jackson Laboratory. To construct macrophage‐conditional RBM25 knockout mice (*Rbm25* ^flox/flox^ *Lyz2* ^cre+^), *Rbm25* ^flox/flox^ mice were intercrossed with *Lyz2* ^cre^ mice. IRF3‐deficient mice were kindly provided by Prof. T. Taniguchi (University of Tokyo, Tokyo, Japan). WT C57BL/6 mice were purchased from Shanghai SIPPR‐BK Laboratory Animal Company (Shanghai, China). All mice were bred under specific pathogen‐free conditions. All animal experiments were performed in accordance with NIH Guide for the Care and Use of Laboratory Animals and approved by the Committee on Ethics of Medicine, Naval Medical University (Shanghai, China).

### 4.2 Animal Models

For the IAV infection model, 8‐week‐old C57BL/6 mice were intranasally inoculated with the PR8 influenza virus strain, followed by daily monitoring of survival rates and body weight alterations over the entire experimental course. In the VSV infection model, each 8‐week‐old mouse was administered an intraperitoneal injection of VSV. Regarding the HSV‐1 infection model, 8‐week‐old mice received intraperitoneal inoculation HSV‐1, with post‐infection survival status and body weight fluctuations evaluated continuously. Viral loads in tissues were quantified by plaque assay or TCID_50_ method.

### 4.3 Cell Culture and Viruses

RAW 264.7 cell line, HEK293T cell line, human hepatoma cell line Huh7, and MDCK cell line were from ATCC. To obtain mouse primary peritoneal macrophages, mice (male or female, 6–8‐week‐old) were injected intraperitoneally with 3% fluid thioglycollate medium (Merck). Three days later, peritoneal lavage fluids were collected and centrifuged. Cells were resuspended with DMEM containing 10% fetal bovine serum (FBS, Gibco) and cultured in plates. Two hours later, nonadherent cells were removed, and the adherent monolayer cells were washed with DMEM and used as peritoneal macrophages. The cells above were cultured in endotoxin‐free DMEM supplemented with 10% FBS (Gibco). Bone marrow‐derived macrophages were generated by cultivating mouse bone marrow cells in RPMI 1640 medium containing 10% FBS (Gibco) supplemented with recombinant M‐CSF (20 ng/mL). Fresh medium was added on day 3, and the fully differentiated macrophages were harvested on day 6 for the function assay.

The IAV strain PR8 (A/Puerto Rico/8/1934, H1N1) was from ATCC (Cat# VR‐1469), the virus was amplified using 9‐day‐old embryonic chicken eggs and then titrated by determining log_10_PFU/ml on MDCK cells or determining total virus particles with the chicken hemagglutination test. HSV‐1 was a gift from Q. Li (Chinese Academy of Sciences, Beijing, China), VSV was a gift from W. Pan (Navy Medical University, Shanghai, China). VSV‐GFP and SEV were kept in our lab, as described previously [38].

### 4.4 Reagents and Antibodies

Poly (I:C) were from Sigma–Aldrich. Protein A/G Plus‐Agarose Immunoprecipitation Reagent (sc‐2003) used for IP was from Santa Cruz Biotechnology. Protease inhibitor cocktail set III (539134) was from Millipore. Antibody against RBM25 was from Novus Biologicals (NB100‐57505). Antibodies against β‐Actin (4967), RIG‐I (3743), MAVS (3993), VSV‐G (81454S), p‐TBK1 (5483S), TBK1 (3504), IRF3 (4302S), p‐STAT1(9167S), STAT1 (9172S), and Rab5 (3547) were from Cell Signaling Technology. Antibodies against IAV HA (sc‐52025), IAV NP (sc‐101352), and HSV‐gB (sc‐56987) were from Santa Cruz Biotechnology. Antibody against p‐IRF3 (MA5‐14947) was from Thermo Scientific. Antibody against F4/80 was from Abcam (ab6640). Antibody against Rab22a (12125‐1‐AP) was from ProteinTech. Antibody against RC3H1 (A300‐514A) was from Bethyl laboratories. The following antibodies for flow cytometry including 7‐AAD (Percp), CD45 (clone: 30‐F11, BV650), CD11c (clone: N418, APC), CD11b (clone: N418, BV510), F4/80 (clone: BM8, FITC), Ly6G (clone: 1A8, APC/Cy7‐Ly6G), Ly6c (clone: HK1.4, AF700), CD4 (clone: GK1.5, BV421) NK1.1 (clone: PK136, PE) and CD8a (clone: 53–6.7, BV510) were from Biolegend. B220 (clone: RA3‐6B2, PE) was from BD Biosciences.

### 4.5 RNA Isolation and Quantitative PCR (RT‐qPCR) Assay

Total RNAs from peritoneal macrophages, BMDMs or lung, liver or brain tissues were extracted using TRIzol reagent (Invitrogen), and reverse‐transcribed into cDNA using the HiScript III All‐in‐one RT SuperMix Perfect for q‐PCR kit (Vazyme) according to the manufacturer's instructions. The cDNA of each sample was used to assess changes in the expression of different genes via LC480 Real‐Time PCR Systems (Roche) and SYBR q‐PCR Master Mix (Vazyme). The q‐PCR data were normalized to mouse *Actb* or human *ACTB* expression. The sequences of the primers are listed in Table S1.

### 4.6 RNA Interference

The specific siRNA oligonucleotide mixture targeting mouse *Rbm25* were from Dharmacon (J‐057045‐05‐0005). The specific siRNA oligonucleotide mixture targeting human *RBM25* were from Santa Cruz (sc‐92256). The specific siRNA targeting human *RC3H1* was from Santa Cruz (sc‐78873). The specific siRNA oligonucleotides targeting mouse *Rab22a* were from GenePharma (Shanghai, China), and the sequence of *Rab22a*‐siRNA was 1#: 5′‐GUGUGGGUAAAUCGAGCAUTT‐3’; 2#: 5′‐CAGCAGCCAUCAUCGUUUATT‐3’; 3#: 5′‐GAGCUACAUAAAUUCCUAATT‐3’. The sequence of Ctrl‐siRNA was 5′‐UUCUCCGAACGUGUCACGUTT‐3’. The specific siRNA oligonucleotides targeting mouse *Rc3h1* was from GenePharma, and the sequence of *Rc3h1*‐siRNA was 1#: 5′‐GGACUUGGCUCAUAAAUCAdTdT‐3’; 2#: 5′‐CCUUCUAUCUGCUGAAAGAdTdT‐3’; 3#: 5′‐CGCACAGUUACAGAGCUCAdTdT‐3’. The delivery of siRNAs into cells was carried out with Lipofectamine RNAiMAX transfection reagent (Invitrogen, Carlsbad, CA) according to the manufacturer's instructions. After 48 h transfection, cells were collected for further analysis.

### 4.7 Plasmid Construction and Transfection

The human RBM25 coding sequence (CDS) was amplified by PCR‐based amplification using human cell‐derived cDNA as the template and subsequently cloned into the p3xFLAG‐CMV‐14 (Sigma). Each construct was confirmed by DNA sequencing. Plasmid transfection was performed using Lipofectamine 3000 reagent according to the manufacturer's instructions, and cells were harvested 48 h post‐transfection for subsequent experiments.

### 4.8 ELISA

IFN‐β levels in the supernatants or sera were measured using a mouse IFN‐β ELISA kit (PBL Biomedical Laboratories) according to the manufacturer's instructions.

### 4.9 Immunoblot and Immunoprecipitation Analysis

Total proteins of cells were extracted with cell lysis buffer (Cell Signaling Technology) and additional protease inhibitor cocktail set III (Millipore #539134) and 1 mm phenylmethylsulphonyl fluoride. The concentrations of the extracted proteins were measured with BCA protein assay kit (Thermo Fisher Scientific). Equal amounts of protein were used to perform immunoblot analysis. Quantifications of specific protein bands were conducted with the ImageJ software. For immunoprecipitation assays, equal amounts of total proteins were incubated overnight at 4°C with the antibody against target protein. Protein‐antibody complexes were collected with protein A/G agarose beads (Santa Cruz). After 2 h of incubation, protein and bead mixtures were centrifuged, washed, and resuspended with protein loading buffer, and then analyzed by immunoblot analysis.

### 4.10 Flow Cytometry Analysis

For the assessment of viral infection, macrophages were infected with VSV‐GFP at a multiplicity of infection (MOI) of 10 for 8 h, followed by thorough rinsing with phosphate‐buffered saline (PBS), detachment via trypsinization, and resuspension in fluorescence‐activated cell sorting buffer prior to analysis on a Flow Cytometer (Agilent NovoCyte). For the assessment of viral penetration, peritoneal macrophages were precooled at 4°C for 2 h and infected with VSV‐GFP at 4°C for 1 h to allow the virus to attach to the cells. The cells were washed with cold PBS and then incubated at 37°C for 1 h to allow VSV‐GFP penetration into cells. The cells were then harvested by trypsin digestion and resuspended in FACS buffer, followed by flow cytometry analysis.

For the assessment of development and differentiation of key antiviral immune cells in *Rbm25*‐cKO and WT mice, single‐cell suspensions were prepared from murine spleen, liver, and PBMCs. Cells were blocked with anti‐CD16/32 (BioLegend) and then stained with fluorochrome‐conjugated antibodies at 4°C for 1 h. The following antibodies were used: Percp‐7AAD, BV650‐CD45, APC‐CD11c, BV510‐CD11b, FITC‐F4/80, APC/Cy7‐Ly6G, AF700‐Ly6c, BV421‐CD4, and BV510‐CD8a and PE‐B220. Data were acquired on a flow cytometer (Agilent NovoCyte) and analyzed with FlowJo software (version 10.10.0).

### 4.11 TCID50 Assay and PFU Assay

To determine the plaque‐forming unit of PR8 influenza virus, viral supernatants were collected, and plaque titers were determined by the plaque assay [39]. Briefly, monolayer MDCK cells in 12‐well plates were incubated with serial dilutions (10 times) of viral supernatants in 0.25 ml at room temperature (RT) for 1 h with swirling every 15 min. One milliliter of 1% agarose with 0.25% fetal bovine serum was then added to the cells and left at RT until it set. Then the dishes were turned upside down and incubated at 37°C. At 72 h post infection, the agarose layer was removed, and the plaques were visualized with 0.1% crystal violet solution.

The semi‐automated version of the TCID50 assay with algorithm‐based evaluation of cytopathic effects (CPEs) was performed as described previously [40]. HEK293T cells (VSV) or VERO cells (HSV‐1) were seeded in 96‐well plates. After 24 h, adherent cells were infected with 8 replicates of a ½‐log10 serial sample dilution. At 72 h post‐infection, bright‐field images were observed. An algorithm determined the cell area in each well. Wells with a cell area below a certain threshold were classified as CPE‐positive. The model of Spearman/Kärber or Reed/Münch [41] was used to calculate the infectious virus titer.

### 4.12 mRNA Decay Measurement by RT‐qPCR Assays

Cells were treated with 5 µg/mL of actinomycin D (Selleck) to block the transcription. At 0, 1, 2, and 4 h post‐actinomycin D treatment, total RNA was harvested using Trizol (Invitrogen) according to the manufacturer's protocol. Abundance of specific RNA was quantified by RT‐qPCR. mRNA levels were normalized against *Actb* (mouse) or *ACTIN* (human) mRNA and plotted against time.

### 4.13 Detection of the Attached and the Penetrated Viruses

The attachment and the penetration of viruses were assayed as previously described [42]. For the detection of viral attachment, the cells were precooled at 4°C for 2 h and then incubated with viruses at 4°C for 1 h. Subsequently, the cells were washed with PBS to remove unbounded viruses and subject to preparation of viral RNA. For the detection of viral penetration, the PBS‐washed cells were immediately moved to incubators and cultured at 37°C for 1 h. Subsequently, the cells were either lysed to detect viral RNA.

### 4.14 Immunofluorescence (IF)

Mouse tissues obtained immediately post‐harvest were immersed in 4% paraformaldehyde embedded, and subsequently sectioned into thin slices. To minimize non‐specific antibody binding, the tissue sections were subjected to blocking treatment in a solution supplemented with 10% goat serum and the non‐ionic detergent Triton X‐100. Thereafter, the sections were incubated overnight at 4°C with a primary antibody targeting the protein of interest, which was pre‐diluted in the aforementioned blocking buffer. Upon completion of primary antibody incubation, the slices were rinsed extensively with phosphate‐buffered saline (PBS). Subsequently, the sections were incubated with fluorophore‐labeled secondary antibodies under light‐protected conditions. After a final washing step, the tissue slices were mounted onto glass slides using a DAPI‐containing mounting medium and visualized under a fluorescence microscope.

### 4.15 Histopathology and Immunohistochemistry (IHC)

Mouse tissues were fixed in 4% paraformaldehyde, embedded in paraffin, and sectioned. Sections were stained with H&E for histological analysis. For immunohistochemistry, sections were incubated with antibodies against viral antigens or cell markers, followed by appropriate secondary antibodies. IHC staining was performed according to standard procedures. In brief, tissue slices were incubated with primary antibody against HSV‐gB at 4°C overnight. After incubation with a secondary antibody for 20 min, the sections were stained with horseradish peroxidase (HRP)‐conjugated streptavidin for 20 min and developed using a DAB solution. The mean density and HSV gB‐positive cells of the corresponding IHC image were calculated by ImageJ software.

### 4.16 RNA‐seq and Data Analysis

RNA‐seq was performed as described before [43]. RNA of the peritoneal macrophages was extracted for RNA‐seq. RNA paired‐end reads of 150 bp from RNA samples were generated with the Illumina NovaSeq 6000. Based on the default parameter settings of HISAT2 [44], the reads were mapped to the reference mouse genome (mm10). Fragment per kilobase of transcript per million mapped reads (FPKM) values were performed to evaluate differential gene expression. The number of counts for each sample gene were normalized using DESeq260, and the multiplicity of differences were calculated. The differentially expressed genes were analyzed by Gene Ontology (GO) functional enrichment analysis and Kyoto Encyclopedia of Genes and Genomes (KEGG). The RNA sequencing data have been deposited in the Gene Expression Omnibus with accession no. GSE314891.

### 4.17 Confocal Microscopy

Macrophages were fixed with 4% paraformaldehyde for 15 min and permeabilized with 0.2% Triton X‐100 for 5 min. After blocking with 5% BSA, cells were labeled with anti‐Rab5 antibody followed by staining with corresponding secondary antibodies. Cells were observed with a Leica TCS SP8 confocal laser microscope.

### 4.18 Statistical Analysis

All statistical analyses were performed using GraphPad Prism (version 10). Statistical significance between different groups was determined by an unpaired two‐tailed Student's *t* test or ANOVA. The statistical significance of survival curves was estimated according to the method of Kaplan and Meier, and the curves were compared with the log‐rank (Mantel‐Cox) analysis. Spearman's rank correlation analysis was performed to evaluate the correlation between two variables. Differences were considered to be significant when *p* < 0.05.

## Author Contributions

Y.D., H.C., Y.J., C.Z., and J.B. performed experiments, analyzed data, and interpreted results. Y.D. designed experiments and drafted the manuscript. Y.X., Z.W., X.W., B.R., W.T., and Y.D. performed experiments and collected data. X.L., Y.Z., and Z.Z conceived the study, designed experiments, analyzed data, and revised the manuscript. X.L. supervised this study. All authors read and approved the manuscript.

## Conflicts of Interest

The authors declare no conflicts of interest.

## Supporting information




**Supporting File 1**: advs76160‐sup‐0001‐SuppMat.pdf.

**Supporting File 2**: advs76160‐sup‐0002‐Data.pdf.

## Data Availability

The RNA‐seq data have been deposited at GEO GSE314891 and the RIP‐seq data have been deposited at GEO GSE240159. The data that support the findings of this study are available from the corresponding author upon reasonable request.
