# Supplementary material for: RNA‐Binding Protein RBM25 Targets the mRNA Stability of GTPase Rab22a to Restrict Viral Entry and Infection
Source: Adv Sci (Weinh). 2026 Jun 16:e76160. Online ahead of print. doi: 10.1002/advs.76160 (PMC13336907; doi:10.1002/advs.76160)
Supplement: Supplementary file 1 — Supporting File 1: advs76160‐sup‐0001‐SuppMat.pdf. [file ADVS-9999-e76160-s002.pdf]

Supporting Information

RNA-binding protein RBM25 Targets the mRNA stability of GTPase Rab22a to Restrict Viral Entry and Infection

Yingying Ding, *Huiying Chen*, *Yuyu Jiang*, *Chunyan Zhao*, *Jie Bai*, *Yan Xiang*, *Zeting Wang*, *Xixi Wang*, *Bing Rui*, *Wanda Tang*, *Yue Ding*, *Zhenzhen Zhan*, *Yunkai Zhang*, *Xingguang Liu*

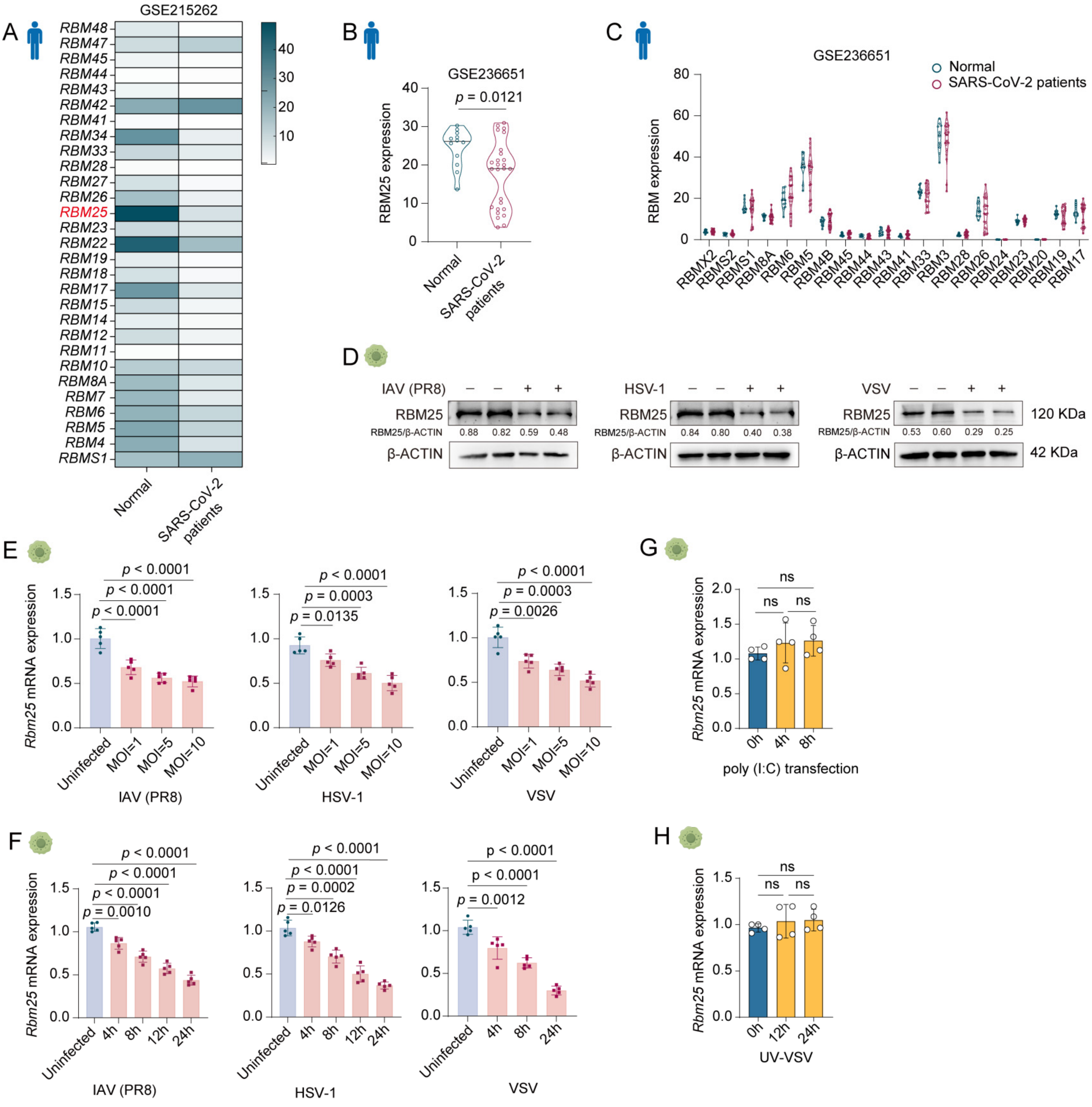

Figure S1. RBM25 expression is decreased following various virus infection.

(A) A heatmap displaying the expression of different RBPs in PBMCs from public microarray datasets (GEO: GSE215262; SARS-CoV-2 infected patients, n = 8; normal controls, n = 15).

(B) Violin-plots of RBM25 expression in PBMCs from public microarray datasets (GEO: GSE236651; SARS-CoV-2 infected patients, $n = 26$; normal controls, $n = 12$).

(C) Violin-plots of the expression of different RBPs except RBM25 in PBMCs from public microarray datasets (GEO: GSE236651; SARS-CoV-2 infected patients, n = 26; normal controls, n = 12).

(D) Immunoblots analysis of RBM25 in mouse peritoneal macrophages infected with influenza virus PR8, HSV-1 or VSV for 8 h or left untreated. β-ACTIN was used as a loading control.

(E) RT-qPCR analysis of *Rbm25* mRNA level in mouse peritoneal macrophages infected with influenza virus PR8, HSV-1 or VSV for 8 h at different MOI (MOI=1, MOI=5 or MOI=10).

(F) RT-qPCR analysis of *Rbm25* mRNA level in mouse peritoneal macrophages infected with influenza virus PR8, HSV-1 or VSV at MOI=1 for the different times (4h, 8h,12h, and 24h).

(G-H) RT-qPCR analysis of *Rbm25* mRNA in WT peritoneal macrophages transfected with poly (I:C) (G) or stimulated with UV-VSV (H) for the indicated times.

Data are presented as the mean ± SD. Statistical significance between two groups was determined by unpaired Student's t-test (C) and one-way ANOVA tests (E-H) .

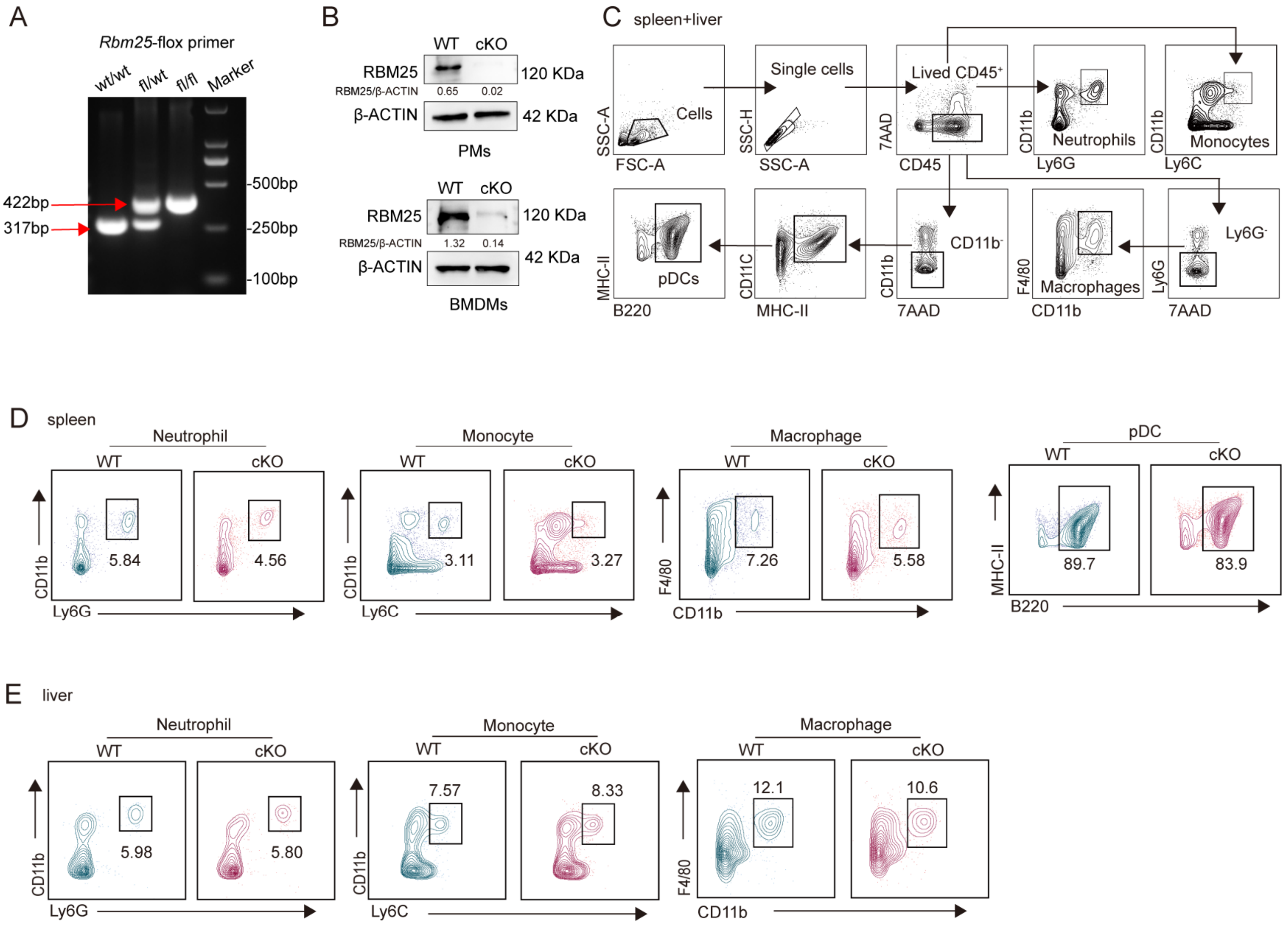

Figure S2. RBM25 deficiency in macrophages does not impair development of neutrophil, monocyte, macrophage and pDC in mice.

(A) Genotyping results of *Rbm25* allele in *Rbm25*-cKO and WT mice.

(B) Immunoblot analysis of RBM25 protein in peritoneal macrophages (PMs) or BMDMs from WT and *Rbm25*-cKO mice.

(C) Schematic of the gating strategy used to identify neutrophils, monocytes, macrophages, and pDCs from mouse spleen and liver by flow cytometry. Representative plots show the sequential gates applied for analysis.

(D-E) Flow cytometry analysis of the levels of neutrophils, monocytes, macrophages, and pDCs in the spleens (D) and livers (E) of WT and *Rbm25*-cKO mice.

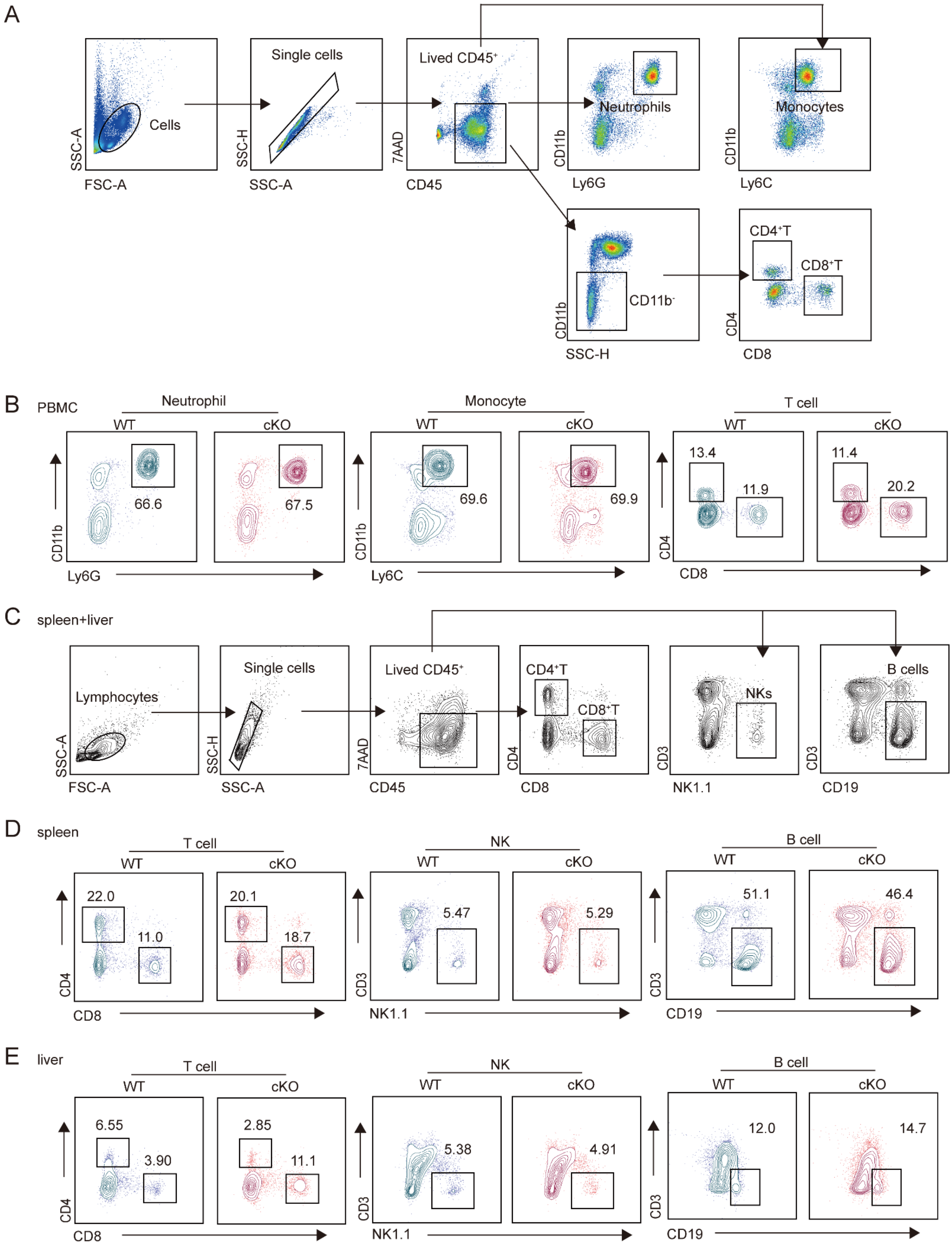
A
Cells
SSC-A
FSC-A
Single cells
SSC-H
SSC-A
Lived CD45+
7AAD
CD45
Neutrophils
CD11b
Ly6G
Monocytes
CD11b
Ly6C
CD11b-
CD11b
SSC-H
CD4+T
CD8+T
CD4
CD8
B
PBMC
Neutrophil
WT
cKO
66.6
67.5
CD11b
Ly6G
Monocyte
WT
cKO
69.6
69.9
CD11b
Ly6C
T cell
WT
cKO
13.4
11.9
11.4
20.2
CD4
CD8
C
spleen+liver
Lymphocytes
SSC-A
FSC-A
Single cells
SSC-H
SSC-A
Lived CD45+
7AAD
CD45
CD4+T
CD8+T
CD4
CD8
NKs
CD3
NK1.1
B cells
CD3
CD19
D
spleen
T cell
WT
cKO
22.0
11.0
20.1
18.7
CD4
CD8
NK
WT
cKO
5.47
5.29
CD3
NK1.1
B cell
WT
cKO
51.1
46.4
CD3
CD19
E
liver
T cell
WT
cKO
6.55
3.90
2.85
11.1
CD4
CD8
NK
WT
cKO
5.38
4.91
CD3
NK1.1
B cell
WT
cKO
12.0
14.7
CD3
CD19

Figure S3. RBM25 deficiency in macrophages does not affect the development of T cells, B cells, or NK cells in mice.

(A) Schematic of the gating strategy used to identify neutrophils, monocytes, $CD4^+$ T cells and $CD8^+$ T cells from mouse PBMCs by flow cytometry. Representative plots show the sequential gates applied for analysis.

(B) Flow cytometry analysis of the levels of neutrophils, monocytes, $CD4^+$ T cells and $CD8^+$ T cells in the PBMCs of WT and *Rbm25*-cKO mice.

(C) Gating strategy used to identify $CD4^+$ T cell, $CD8^+$ T cells, NK cells and B cells from mouse spleen and liver by flow cytometry. Representative plots show the sequential gates applied for analysis.

(D-E) Flow cytometry analysis of the levels of $CD4^+$ T cell, $CD8^+$ T cells, NK cells and B cells in the spleens (D) and livers (E) of WT and *Rbm25*-cKO mice.

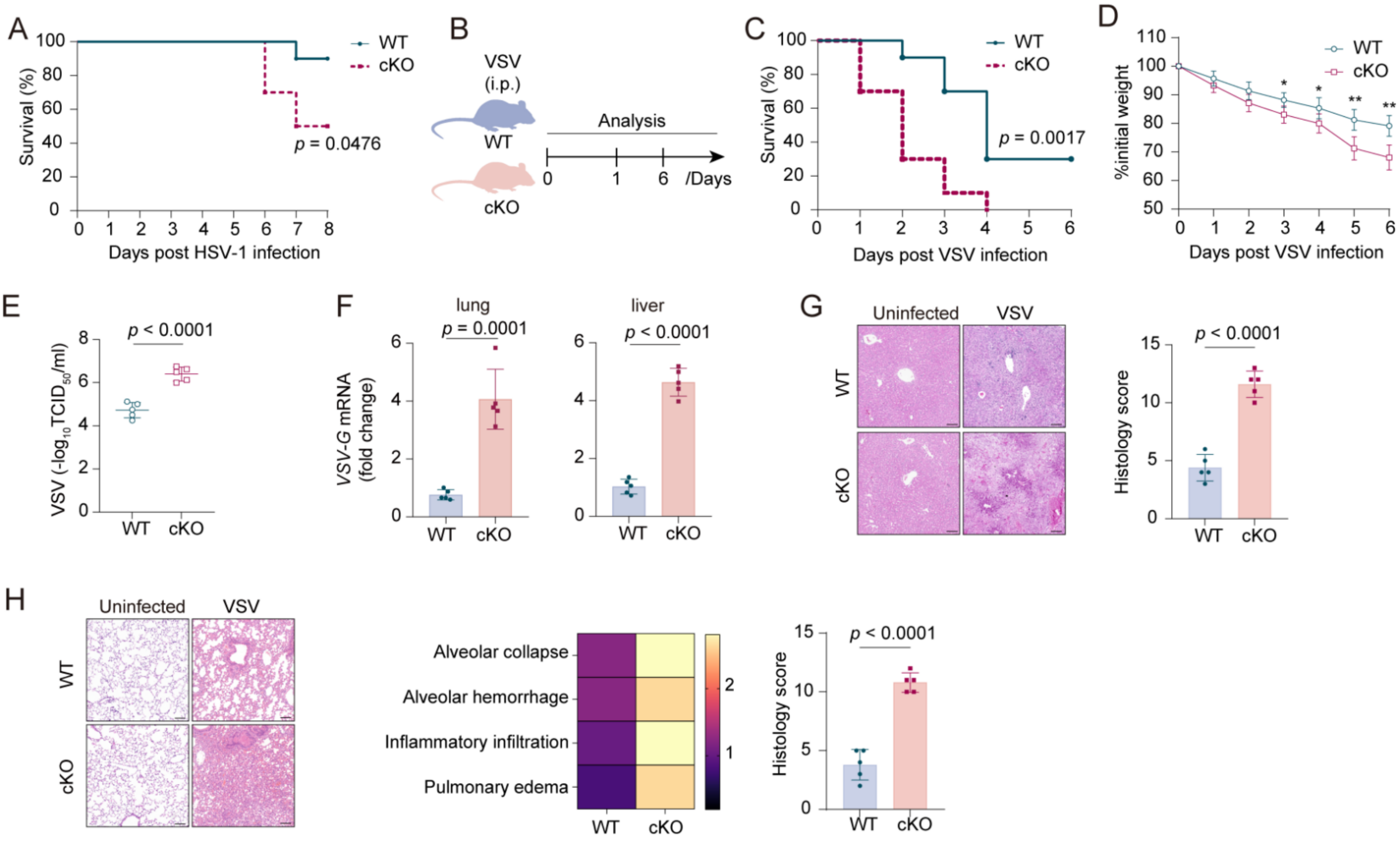

Figure S4. RBM25 deficiency increases the susceptibility to HSV-1 and VSV infection.

(A) Survival of *Rbm25*-cKO and WT mice infected with HSV-1 (n=10 per group).

(B) *Rbm25*-cKO and WT mice were *i.p.* infected with VSV.

(C) Survival of *Rbm25*-cKO and WT mice infected with VSV (1×10^8 PFU per gram body weight, n=10 per group).

(D) Body weight of *Rbm25*-cKO and WT mice infected with VSV (5×10^7 PFU per gram body weight) were monitored over time (n = 5 per group).

(E) VSV titers in the livers from *Rbm25*-cKO and WT mice 24 h after *i.p.* injection of VSV (5×10^7 PFU per gram body weight) (n = 5 per group).

(F) RT-qPCR analysis of *VSV G* mRNA in the lung or liver tissues from mice as in (E).

(G-H) HE staining and histology scoring of the livers (G) and lungs (H) from *Rbm25*-cKO and WT mice (n = 5 per group) as in (E). scale bars, 100 μm.

Data are presented as the mean ± SD. Unpaired two-tailed Student's t-test (D-H). Kaplan-Meier survival curves were compared using log-rank (Mantel-Cox) analysis (A and C).

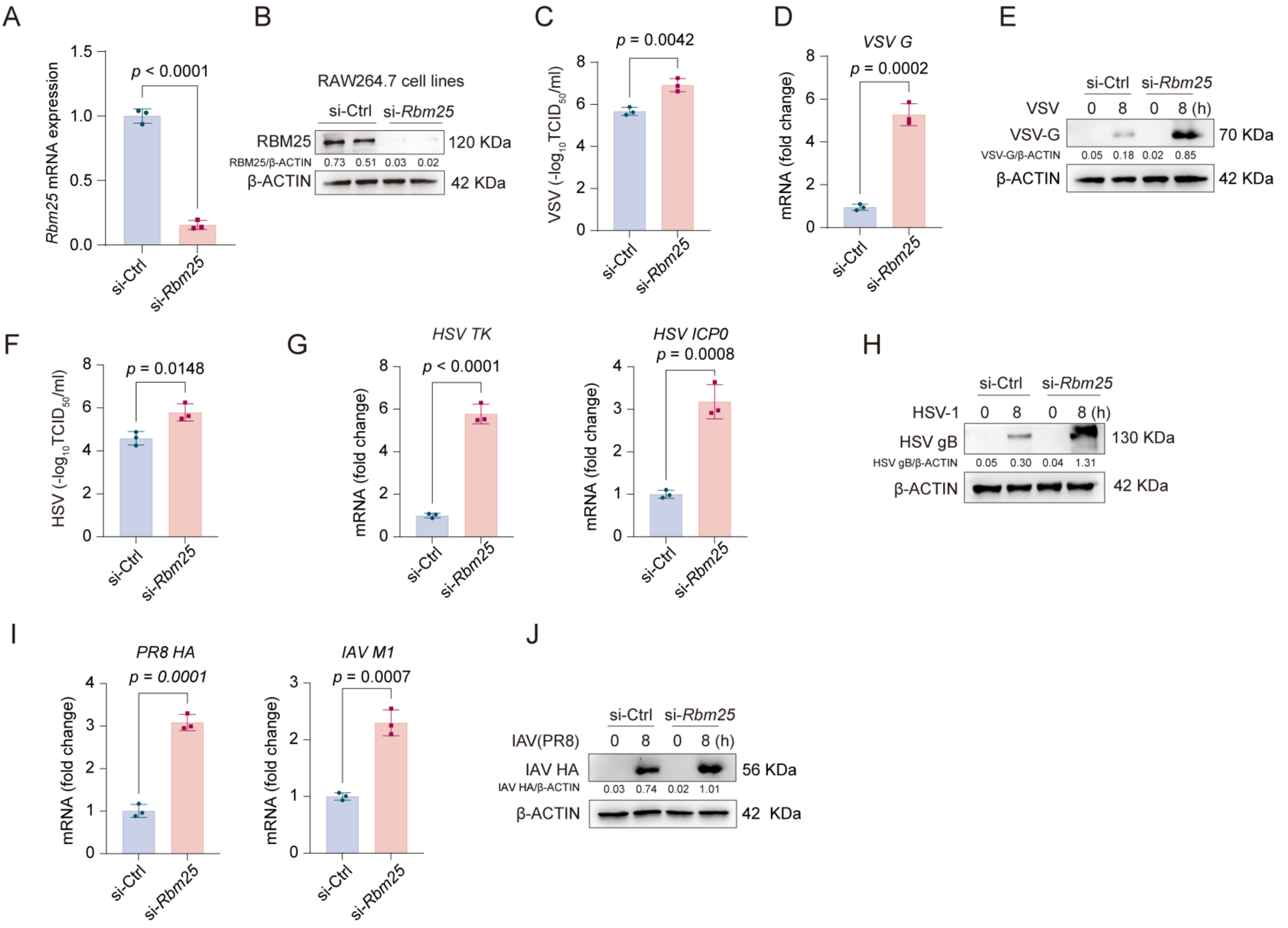

Figure S5. Knockdown of RBM25 facilitates virus replication in RAW 264.7 cells.

(A-B) RT-qPCR analysis (A) and immunoblot analysis (B) of RBM25 expression in RAW 264.7 cells transfected with control siRNA (si-Ctrl) or *Rbm25* siRNA (si-*Rbm25*).

(C) VSV titers in the supernatants of RAW 264.7 cells treated as in (A) and then infected with VSV for 12 h.

(D) RT-qPCR analysis of *VSV G* mRNA in RAW 264.7 cells treated as in (A) and then infected with VSV for 8 h.

(E) Immunoblots analysis of VSV G protein of RAW 264.7 cells treated as in (A) and then infected with VSV for 8 h or not.

(F) HSV-1 titers in the supernatants of RAW 264.7 cells treated as in (A) and then infected with HSV-1 for 24 h.

(G) RT-qPCR analysis of *HSV TK* and *ICP0* mRNA in RAW 264.7 cells treated as in (A) and then infected with HSV-1 for 8 h.

(H) Immunoblots analysis of HSV-1 gB protein in RAW 264.7 cells treated as in (A) and then

infected with HSV-1 for 8 h or not.

(I) RT-qPCR analysis of *IAV HA* and *M1* mRNA of RAW 264.7 cells treated as in (A) and then infected with influenza virus PR8 for 8 h.

(J) Immunoblots analysis of IAV HA protein of RAW 264.7 cells treated as in (A) and then infected with influenza virus PR8 for 8 h or not.

Data are presented as the mean ± SD. Unpaired two-tailed Student's t-test (A, C, D, F, G and I).

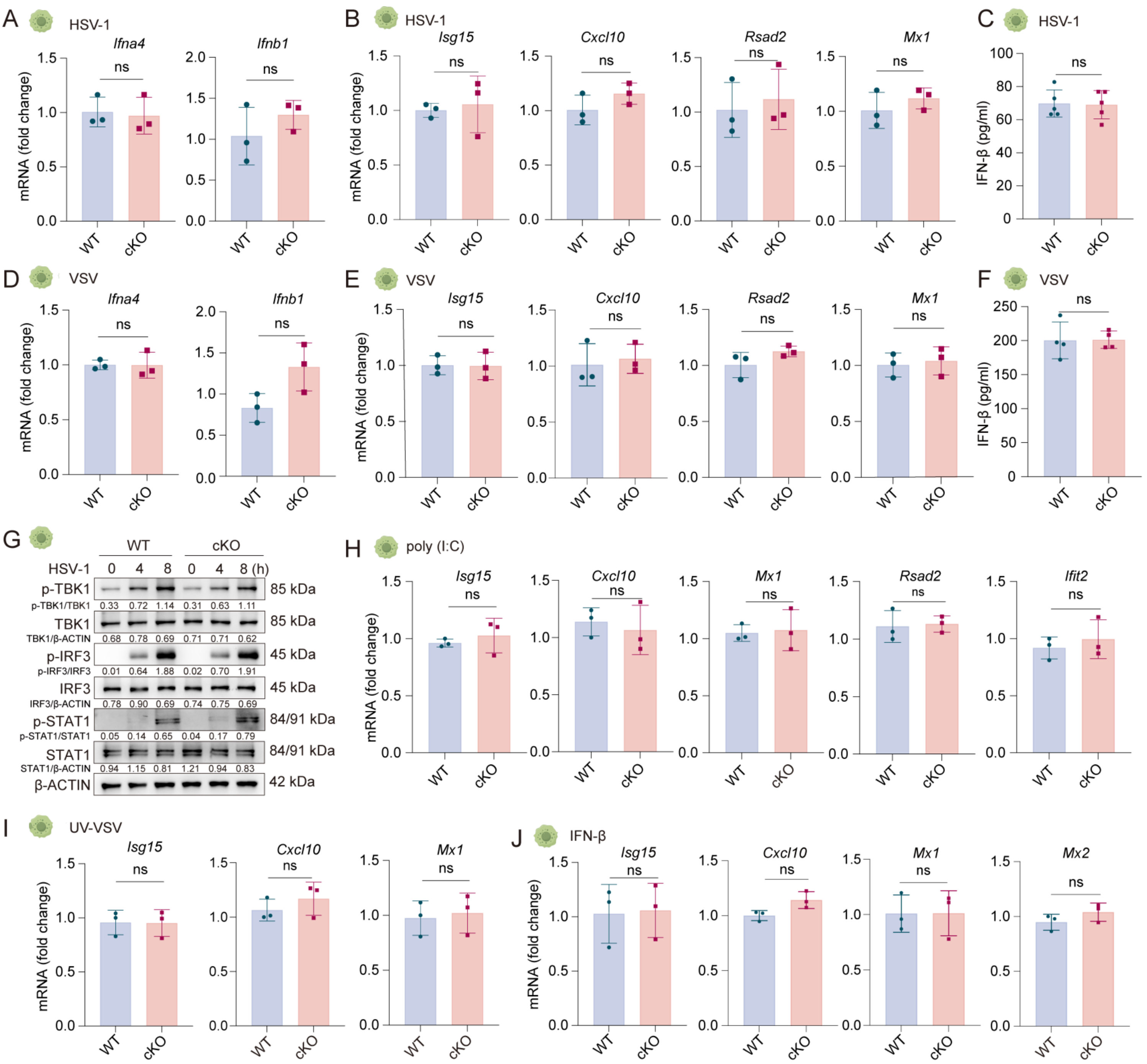

Figure S6. RBM25 barely affects the IFN-I and interferon-stimulated genes expression upon viral infection.

(A-B) RT-qPCR analysis of IFN-I (*Ifna4, Ifnb1*) mRNA (A) and *Isg15*, *Cxcl10*, *Rsad2* and *Mx1* mRNA (B) in WT and *Rbm25*-cKO peritoneal macrophages infected with HSV-1 for 12 h.

(C) ELISA of IFN-β in supernatants of WT and *Rbm25*-cKO peritoneal macrophages infected with HSV-1 for 12 h.

(D-E) RT-qPCR analysis of IFN-I (*Ifna4, Ifnb1*) mRNA (D) and *Isg15*, *Cxcl10*, *Rsad2* and *Mx1* mRNA (E) in WT and *Rbm25*-cKO peritoneal macrophages infected with VSV for 8 h.

(F) ELISA of IFN-β in supernatants of WT and *Rbm25*-cKO peritoneal macrophages infected with VSV for 8 h.

(G) Immunoblots analysis of p-TBK1, TBK1, p-IRF3, IRF3, p-STAT1 and STAT1 in WT and *Rbm25*-cKO peritoneal macrophages infected with HSV-1 for the indicated times.

(H) RT-qPCR analysis of *Isg15, Cxcl10, Mx1, Rsad2* and *Ifit2* mRNA in WT and *Rbm25*-cKO peritoneal macrophages transfected with poly (I:C) for 4 h.

(I) RT-qPCR analysis of *Isg15, Cxcl10* and *Mx1* mRNA in WT and *Rbm25*-cKO peritoneal macrophages treated with UV-VSV for 18 h.

(J) RT-qPCR analysis of *Isg15, Cxcl10, Mx1* and *Mx2* mRNA in WT and *Rbm25*-cKO peritoneal macrophages treated with IFN-β (100 ng/ml) for 8 h.

Data are presented as the mean ± SD. Unpaired two-tailed Student's t-test (A-F and H-J).

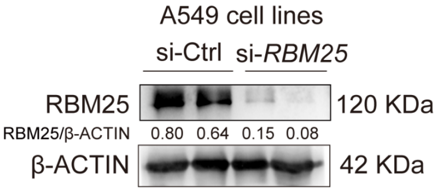

Figure S7. Knockdown efficiency of RBM25 in A549 cells.

Immunoblot analysis of RBM25 expression in A549 cells transfected with control siRNA (si-Ctrl) or *RBM25* siRNA (si-*RBM25*).

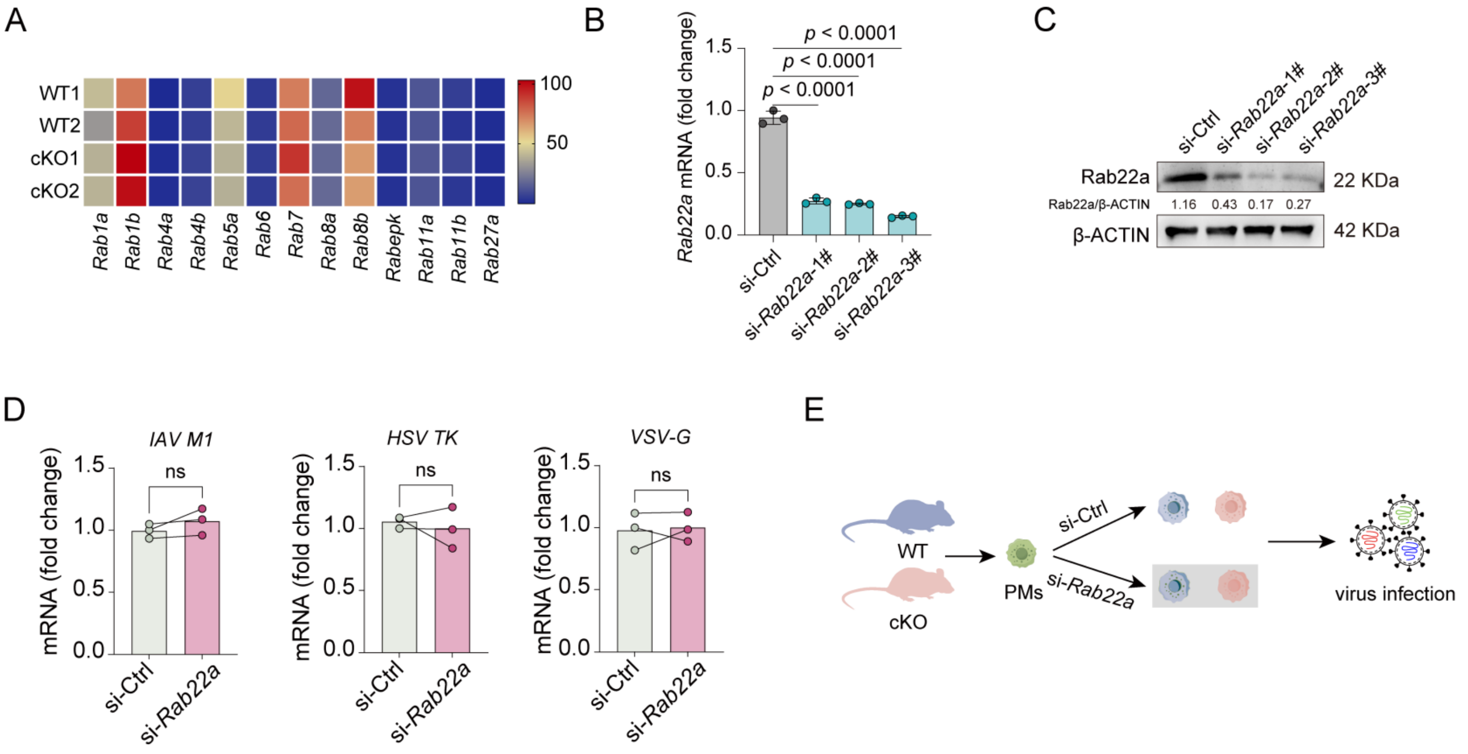

Figure S8. Knockdown of Rab22a does not affect viral attachment of IAV, HSV-1 and VSV.

(A) Heatmap of the expression profiles (FPKM) of Rab genes associated with viral infection based on our previously established RNA-seq dataset.

(B-C) RT-qPCR analysis (B) and immunoblot analysis (C) of Rab22a expression in peritoneal macrophages transfected with control siRNA (si-Ctrl) or three separate *Rab22a* siRNA (si-*Rab22a)*.

(D) RT-qPCR analysis of *IAV M1*, *HSV TK* or *VSV G* mRNA of the attached PR8 influenza virus, HSV-1 or VSV virus in peritoneal macrophages treated as in (B).

(E) A scheme of experiment analyzing the infection of virus in peritoneal macrophages from WT and *Rbm25*-cKO mice transfected with control siRNA or siRNA targeting *Rab22a*.

Data are presented as the mean ± SD. One-way ANOVA test (B), unpaired two-tailed Student's t-test (D).

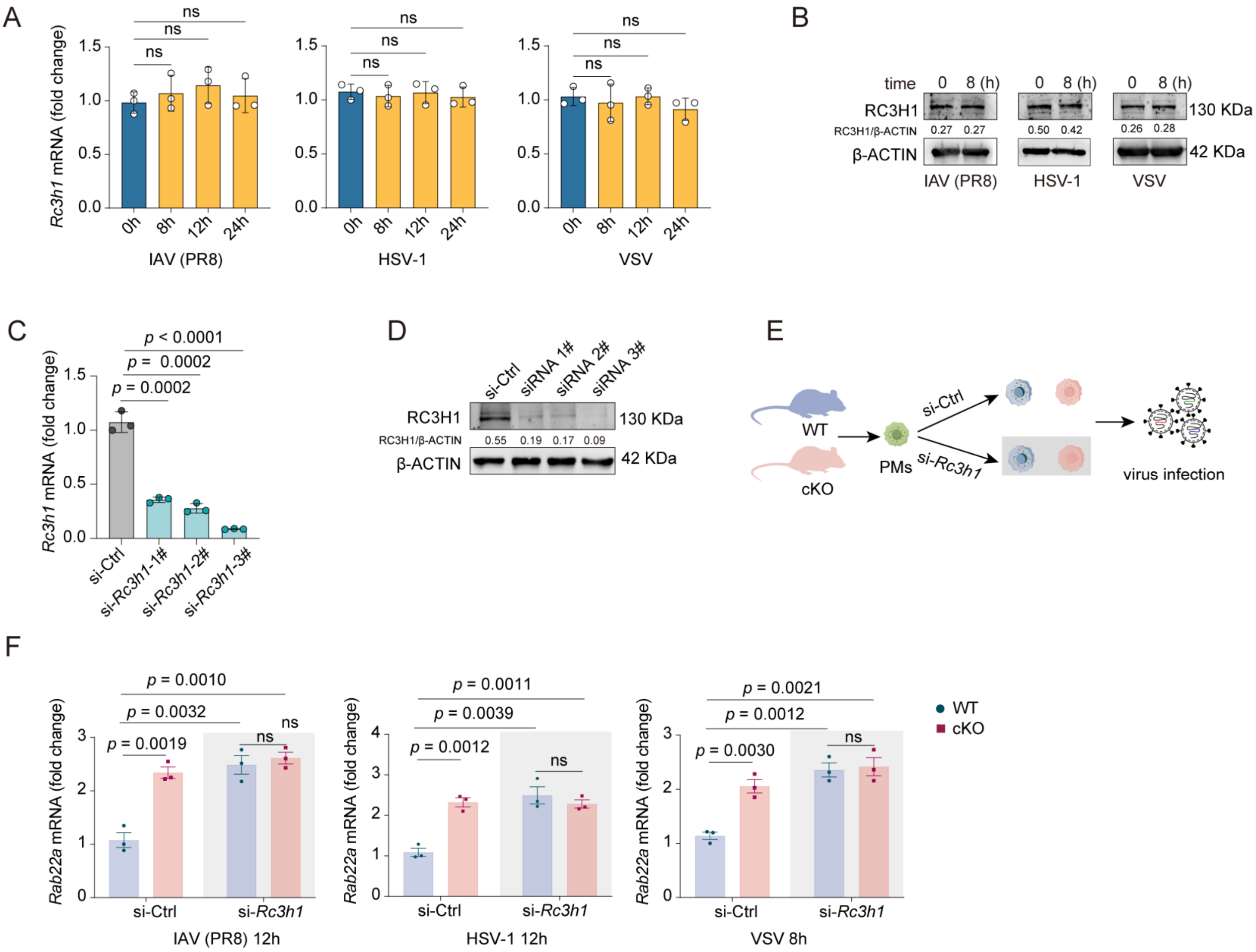

Figure S9. RBM25 inhibits Rab22a expression via RC3H1.

(A-B) RT-qPCR analysis of *Rc3h1* mRNA (A) or immunoblot analysis of RC3H1 protein (B) in WT peritoneal macrophages infected with influenza virus PR8, HSV-1 or VSV for the indicated times.

(C-D) RT-qPCR analysis (C) and immunoblot analysis (D) of RC3H1 expression in peritoneal macrophages transfected with control siRNA (si-Ctrl) or three *Rc3h1* siRNA (si-*Rc3h1)*.

(E) A scheme of experiment analyzing the infection of viruses in peritoneal macrophages from WT and *Rbm25*-cKO mice transfected with control siRNA or siRNA targeting *Rc3h1*.

(F) RT-qPCR analysis of *Rab22a* mRNA in WT and *Rbm25*-cKO peritoneal macrophages transfected with control siRNA or siRNA targeting *Rc3h1* and then infected with the indicated viruses.

Data are presented as the mean ± SD. One-way ANOVA test (A, C), two-way ANOVA test (F).

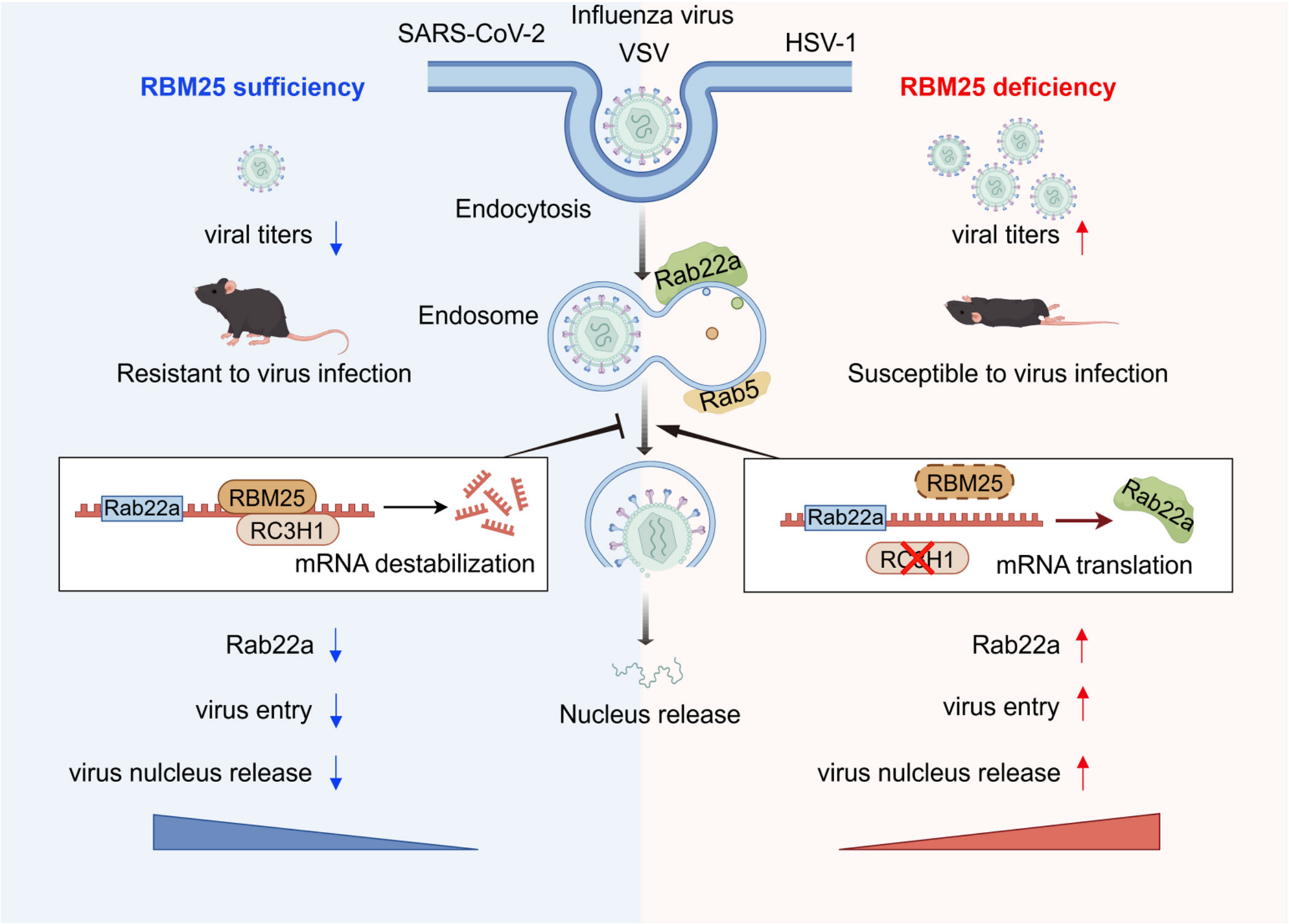

Figure S10. The working model of RBM25 as a gatekeeper for virus entry through destabilizing the mRNA encoding endocytic GPTase Rab22a.

By forming a complex with RC3H1 to bind *Rab22a* mRNA, RBM25 promotes *Rab22a* mRNA degradation and reduces Rab22a expression, thereby suppressing Rab22a-dependent viral entry. This study reveals the RBM25/RC3H1-Rab22a axis as a promising target for the development of host-directed broad-spectrum antiviral strategy.

Table S1. Primers used for RT-qPCR assays.

Name	Forward primer (5'-3')	Reverse primer (5'-3')
Rbm25	GATCCCACCCCCACAGTTTC	CACGGTAGGTACTAGGACAGTT
PR8 HA	AAAGAAAGCTCATGGCCCAACC	TCCTTCTCCGTCAGCCATAGCA
IAV M1	TCAGGCCCCCTCAAAGCCGA	GGGCACGGTGAGCGTGAACA
HSV TK	ACCCGCTTAACAGCGTCAACA	CCAAAGAGGTGCGGGAGTTT
HSV ICP0	AGCGAGTACCCGCCGGCCTG	CAGGTCTCGGTCGCAGGGAAAC
VSV-G	ACGGCGTACTTCCAGATGG	CTCGGTTCAAGATCCAGGT
YFV	GTTCCACAAGGACGCACAAC	TGATCCGCACAGCTTGTCTT
SEV NP	TGCCCTGGAAGATGAGTTAG	GCCTGTTGGTTTGTGGTAAG
Ifna4	TACTCAGCAGACCTTGAACCT	CAGTCTTGGCAGCAAGTTGAC
Ifnb1	ATGAGTGGTGGTTGCAGGC	TGACCTTTCAAATGCAGTAGATTCA
Cxcl10	CCTATGGCCCTCATTCTCAC	CTCATCCTGCTGGGTCTGAG
Mx1	GACCATAGGGGTCTTGACCAA	AGACTTGCTCTTTCTGAAAAGCC
Isg15	GGTGTCCGTGACTAACTCCAT	TGGAAAGGGTAAGACCGTCCT
Mx2	GAGGCTCTTCAGAATGAGCAAA	CTCTGCGGTCAGTCTCTCT
Rsad2	AGCATTAGGGTGGCTAGATCC	CTGAGTGCTGTTCCCATCTTC
Ifit1	GCCTATCGCCAAGATTTAGATGA	TTCTGGATTTAACCGGACAGC
Ifit2	GGAGAGCAATCTGCGACAG	GCTGCCTCATTTAGACCTCTG
Rab22a	GCGCTGAGGGAACTTAAAGTG	ATGCCCCTATGGTTGGATTGA
Rc3h1	TTGTACCTGAAGCCACTCAGCAGT	TCCACTAGCTGGCAATGAACCAGA
Actb	AGTGTGACGTTGACATCCGT	GCAGCTCAGTAACAGTCCGC
RAB22A (human)	CTGGACAAGAACGATTTCGTGC	CATGCTGTCGAAGCTCTTTCA
ACTB (human)	GGCGGCACCACCATGTACCCT	AGGGGCCGGACTCGTCATACT
