# Supplementary material for: RNA‐Binding Protein RBM25 Targets the mRNA Stability of GTPase Rab22a to Restrict Viral Entry and Infection
Source: Adv Sci (Weinh). 2026 Jun 16:e76160. Online ahead of print. doi: 10.1002/advs.76160 (PMC13336907; doi:10.1002/advs.76160)

Figure 1E

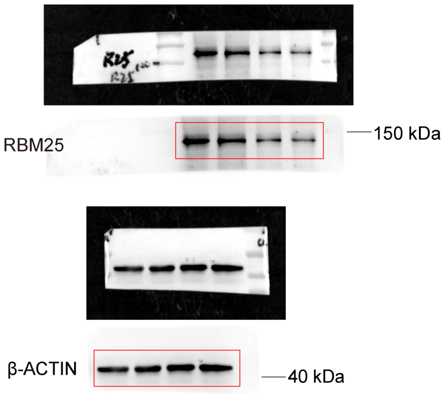

Figure 1F

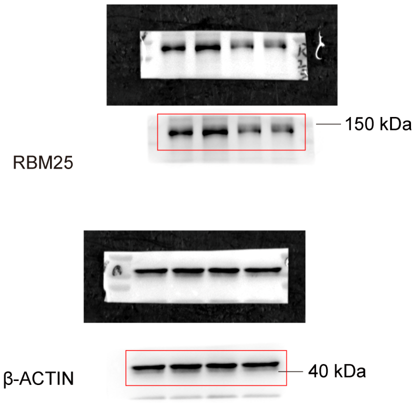

Figure 3C

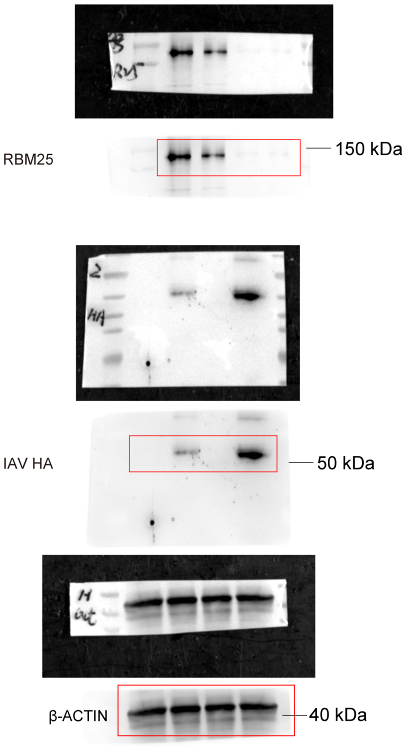

Figure 3G

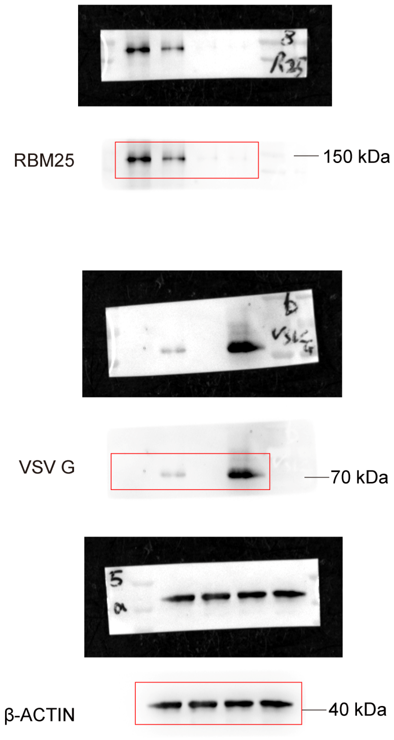

Figure 3L

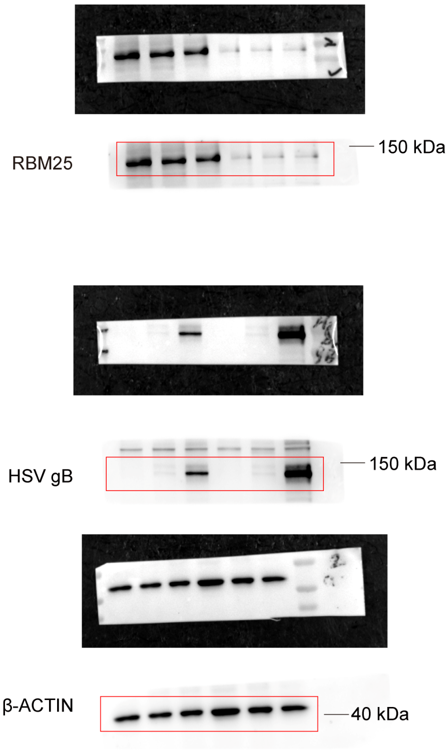

Figure 3O

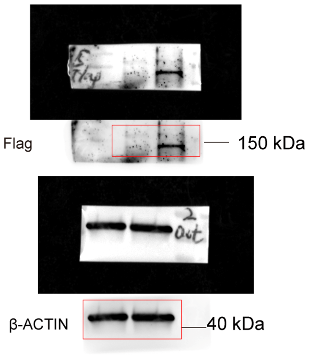

Figure 4I

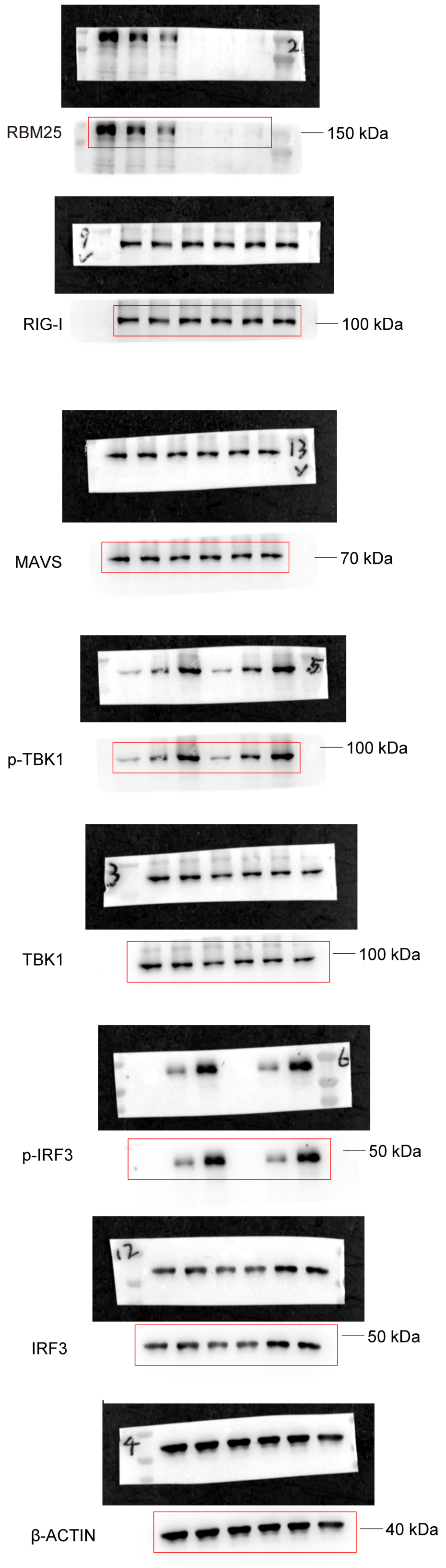

Figure 4N

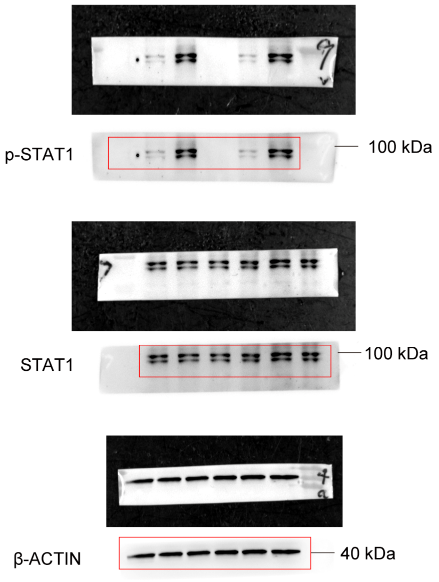

Figure 6H

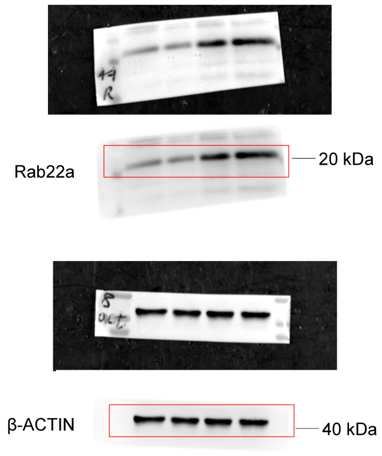

IAV (PR8)

Figure 6H

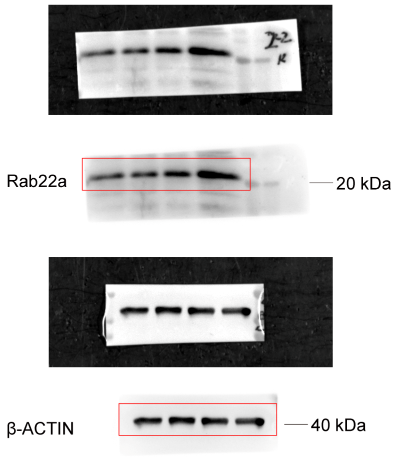

HSV-1

Figure 6H

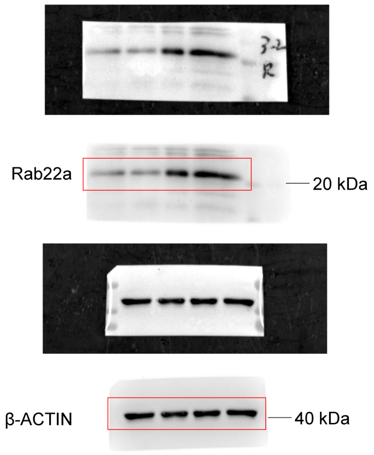

VSV

Figure 6J

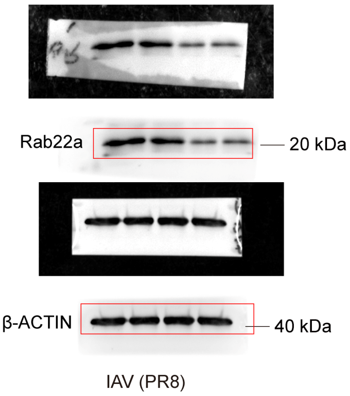

Figure 6J

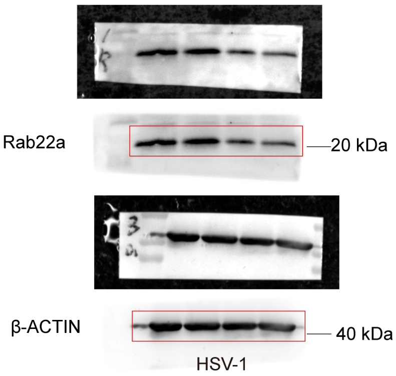

Figure 6J

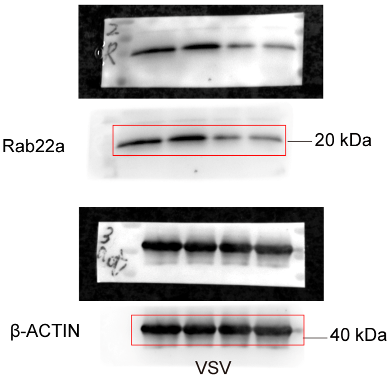

Figure 7D

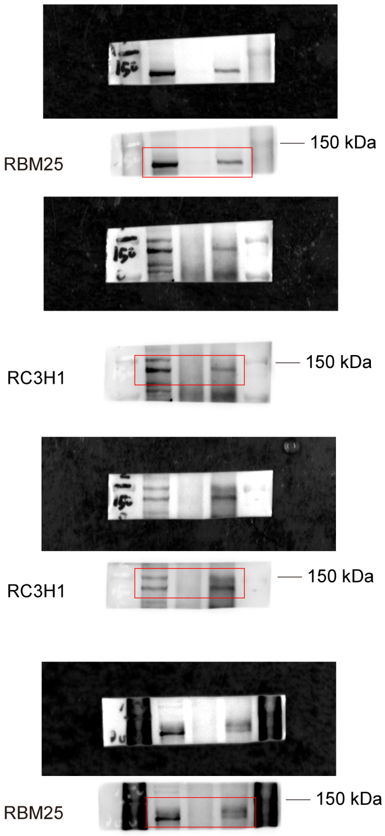

Figure 7E

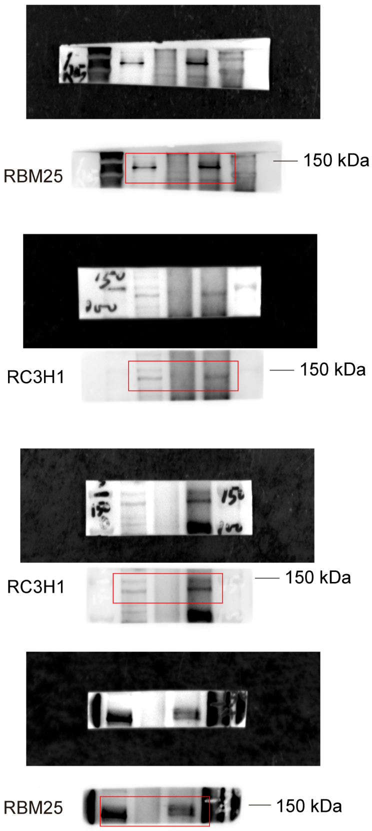

Figure 7I

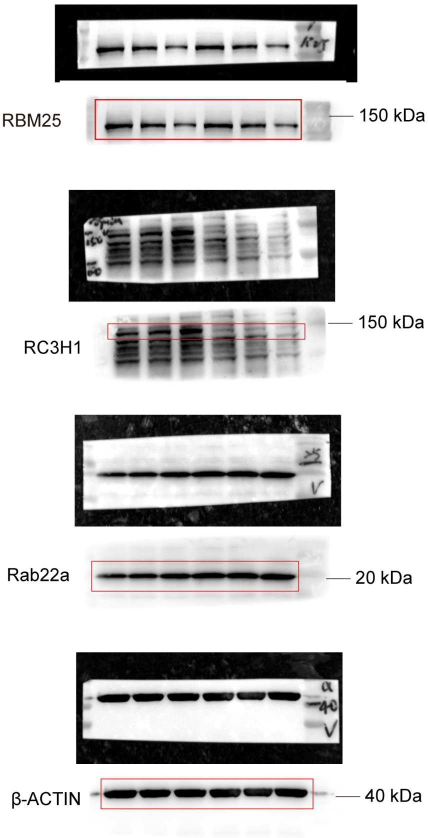

Figure 7J

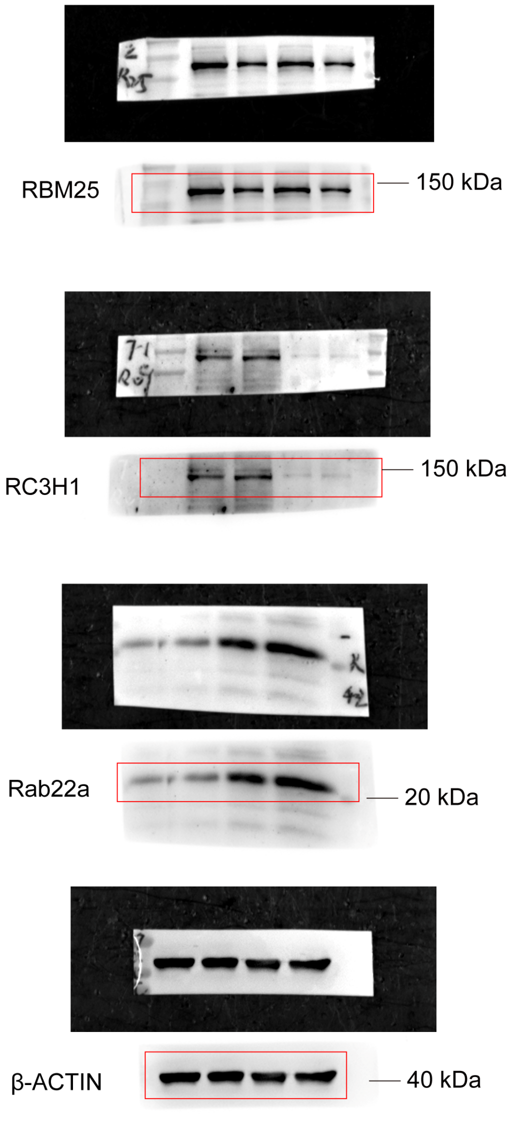

Figure 7N

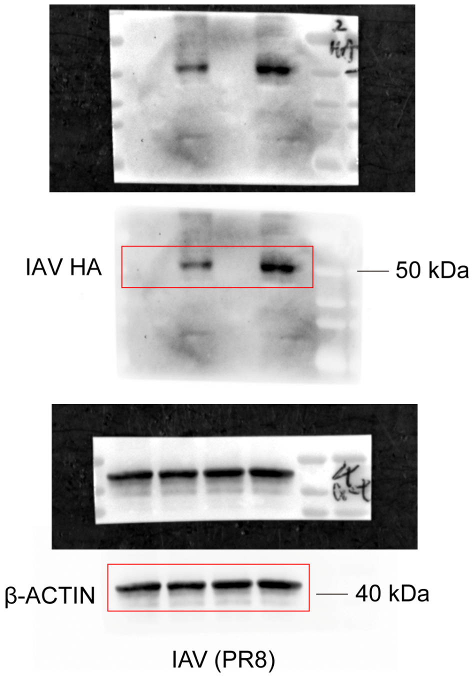

Figure 7N

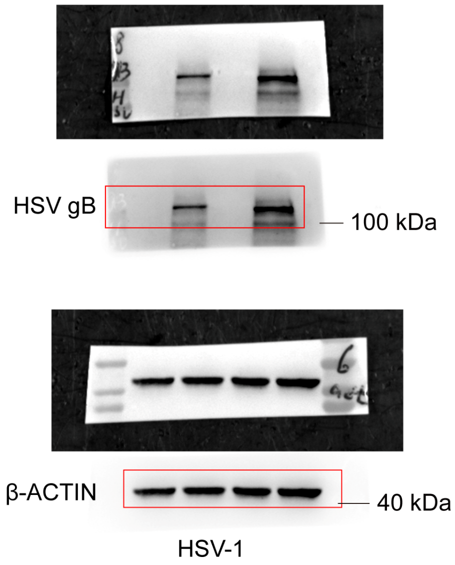

Figure 7N

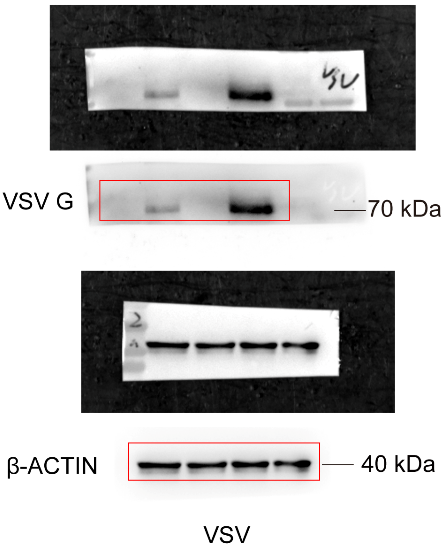

Figure S1D

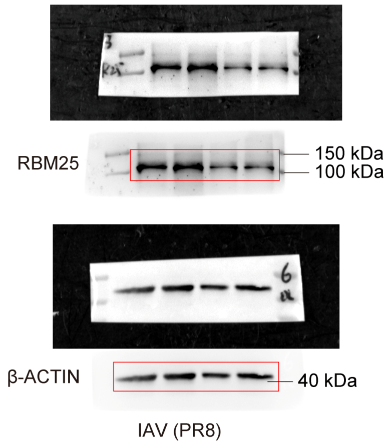

Figure S1D

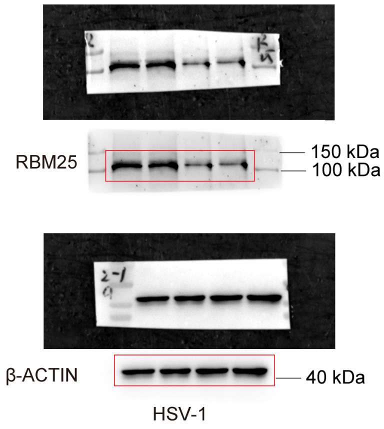

Figure S1D

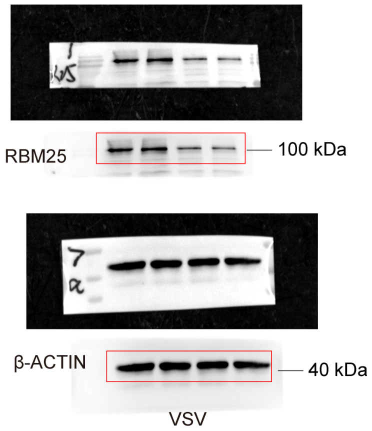

Figure S2A

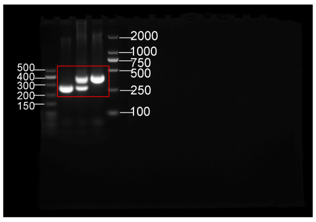

Figure S2B

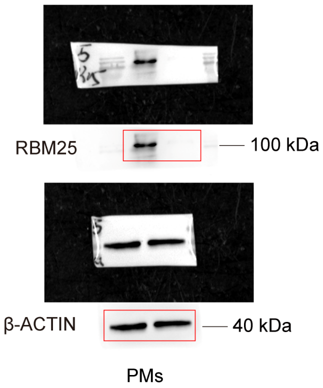

Figure S2B

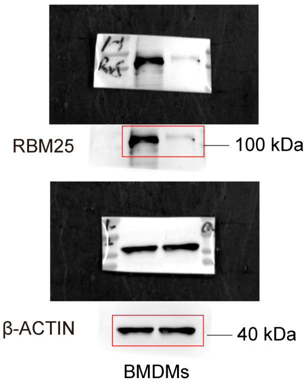

Figure S5B

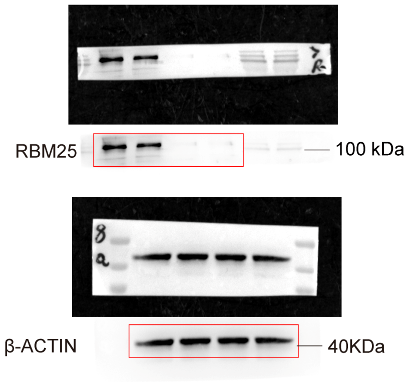

Figure S5H

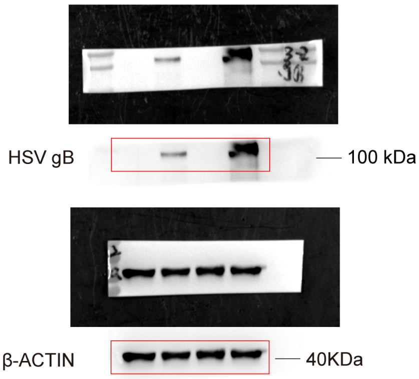

Figure S5J

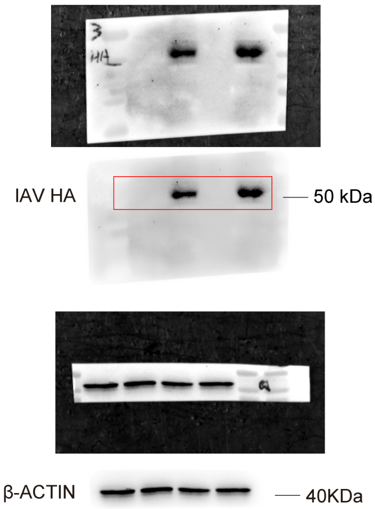

Figure S5E

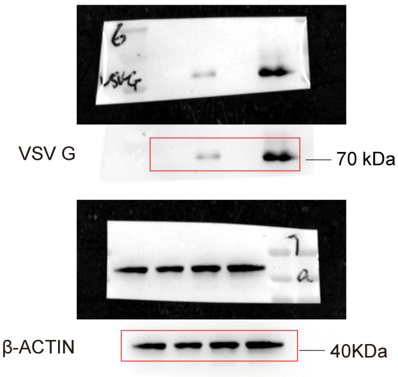

Figure S6G

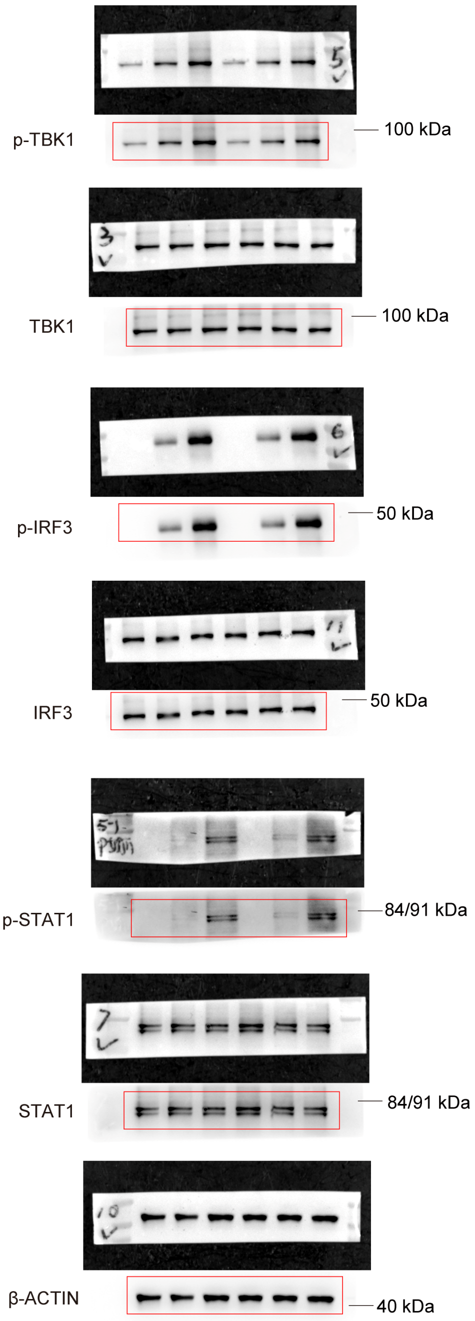

Figure S7

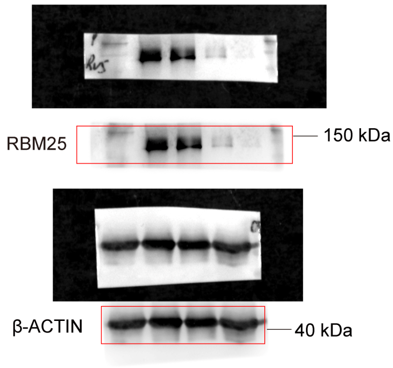

Figure S8C

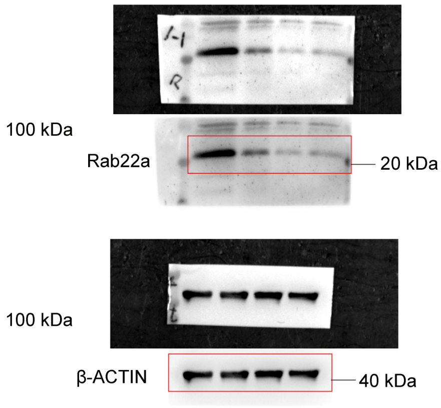

Figure S9B

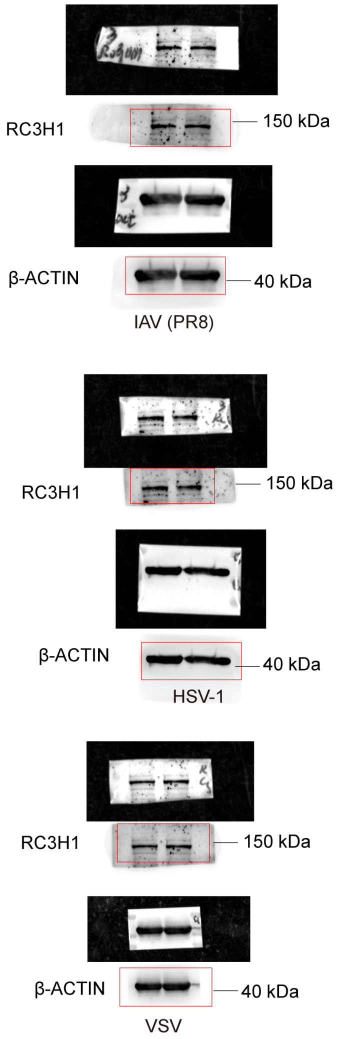

Figure S9D

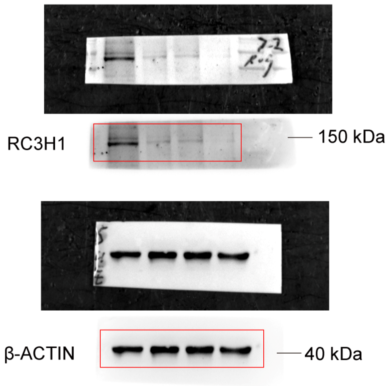

Supplement: Supplementary file 2 — Supporting File 2: advs76160‐sup‐0002‐Data.pdf. [file ADVS-9999-e76160-s001.pdf]
